# Proof-of-principle studies on a strategy to enhance nucleotide imbalance specifically in cancer cells

**DOI:** 10.1038/s41420-022-01254-4

**Published:** 2022-11-24

**Authors:** Twana Alkasalias, Juan Zhang, Harsha Madapura, Basile Dalarun, Oscar Bedoya Reina, Rolf Lewensohn, Kristina Viktorsson, Abbas Salihi, Suhas Darekar, Sonia Laín

**Affiliations:** 1 Department of Microbiology, Tumor and Cell Biology (MTC), Biomedicum, Karolinska Institutet, Stockholm, Sweden; 2 General Directorate of Scientific Research Center, Salahaddin University-Erbil, Erbil, Kurdistan Region Iraq; 3 Science for Life Laboratory, Stockholm, Sweden; 4 Department of Oncology-Pathology, BioClinicum, Karolinska Institutet, Stockholm, Sweden; 5 Theme Cancer, Patient Area Head and Neck, Lung and Skin, Karolinska University Hospital, Stockholm, Sweden; 6 Department of Biology, College of Science, Salahaddin University‑Erbil, Erbil, Kurdistan Region Iraq

**Keywords:** Targeted therapies, Cancer metabolism

## Abstract

Highly specific and potent inhibitors of dihydroorotate dehydrogenase (DHODH), an essential enzyme of the de novo pyrimidine ribonucleotide synthesis pathway, are in clinical trials for autoimmune diseases, viral infections and cancer. However, because DHODH inhibitors (DHODHi) are immunosuppressants they may reduce the anticancer activity of the immune system. Therefore, there may be a need to improve the therapeutic index of DHODHi in cancer patients. The aim of this study was to find strategies to protect activated T cells from DHODHi and to identify cancer types hypersensitive to these inhibitors. First, we observed that like uridine supplementation, adding cytidine to the culture medium protects T cells from DHODH blockage. Next, we identified tumor types with altered expression of pyrimidine ribonucleotide synthesis enzymes. In this regard, we detected that the expression of cytidine deaminase (CDA), which converts cytidine into uridine, is low in an important proportion of cancer cell lines and consistently low in neuroblastoma samples and in cell lines from neuroblastoma and small cell lung carcinoma. This suggested that in the presence of a DHODHi, an excess of cytidine would be deleterious for low CDA expressing cancer cell lines. We show that this was the case (as could be seen almost immediately after treatment) when cells were cultured with fetal bovine serum but, was significantly less evident when cultures contained human serum. One interesting feature of CDA is that aside from acting intracellularly, it is also present in human plasma/serum. Altogether, experiments using recombinant CDA, human serum, pharmacologic inhibition of CDA and T cell/cancer cell co-cultures suggest that the therapeutic index of DHODHi could be improved by selecting patients with low-CDA expressing cancers in combination with strategies to increase cytidine or the cytidine/uridine ratio in the extracellular environment. Collectively, this proof-of-principle study warrants the discovery of agents to deplete extracellular CDA.

## Introduction

Uridine monophosphate (UMP) is the central molecule in pyrimidine nucleotide metabolism [1]. UMP can give rise to UTP, CTP and thymine nucleotides, which in turn are precursors for UDP-sugars, phospholipids, RNA and DNA (Fig. S1). Hence, low levels or imbalance between pyrimidine nucleotides should impair cell growth and if cells fail to arrest in G1, cause stress during DNA replication. In mammals, UMP is generated de novo from glutamine and aspartic acid as well as from plasma pyrimidine nucleosides by the salvage pathway.

Our research group became interested in pyrimidine nucleotide metabolism after carrying out a cell-based screen for p53 activating compounds that unlike the mdm2 inhibitors, would not stop cells in G2, a feature that promotes genomic instability in normal and cancer cells [2]. Surprisingly, a remarkable proportion of these activators inhibited DHODH, a key enzyme in the de novo synthesis of UMP [2–4]. Numerous high quality DHODH inhibitors have been recently described. Several of them are orally available and have antitumor activity in murine cancer models with no reported toxicity [1, 5–9]. Here we have focused on a difference between mice and humans that could be important for the translation of preclinical results with DHODH inhibitors into the clinic. Uridine and cytidine levels in the plasma/sera of mice are in the 1.2–5 μM and in the 1.5–3.6 μM range, respectively. These values are different in plasma from humans where uridine and cytidine are in the 3–5 μM and 0.3–0.7 μM range, respectively [10–13]. These measurements show that cytidine levels and the cytidine/uridine ratio can be higher in murine plasma than in human plasma.

Proliferating T cells are known to be very vulnerable to DHODH inhibitors. Accordingly, the DHODHi leflunomide and its active metabolite teriflunomide have been used for decades against autoimmune diseases [14]. However, immunosuppression may constitute a problem when repurposing these agents for cancer treatment. Therefore, to improve the therapeutic index of DHODHi in cancer patients it is important to identify strategies to rescue activated T cells from DHODH blockage. This study shows that as previously known for extracellular uridine, increasing extracellular cytidine protects activated T cells. It also shows that tumor cell lines expressing low levels of the cytidine deaminase CDA (which converts cytidine into uridine) are hypersensitive to DHODHi in conditions where extracellular cytidine is added in sufficient excess. Altogether, this study indicates that reducing extracellular CDA activity (CDA is not only present inside cells but also in human plasma) may provide a means to increase the therapeutic index of DHODHi especially in patients with cancers where CDA expression is low.

## Results

### Cytidine rescues activated T cells from DHODH inhibition

**Fig. 1:**
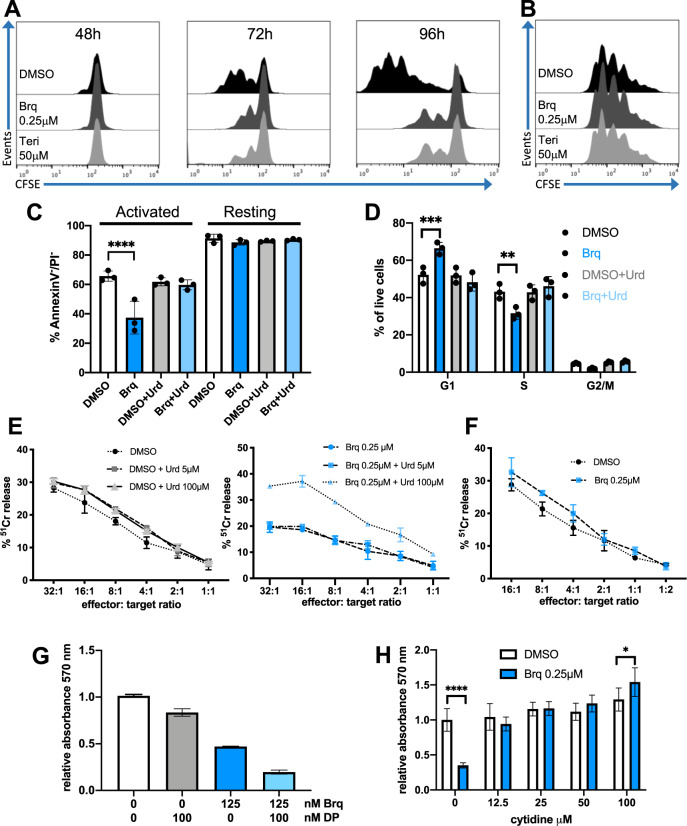
As described for uridine supplementation, addition of cytidine rescues activated T cells from brequinar. **A** T cell division profiles determined by CFSE label dilution. T cells were labelled with CFSE prior to activation by anti-CD3/CD28 antibodies and simultaneously treated with vehicle (DMSO) or DHODH inhibitors brequinar or teriflunomide for 48, 72 and 96 h. CFSE stained cells were analysed by flow cytometry. Representative experiment of 2 biological repeats. (B) Resting T cells were labelled with CFSE prior to the treatment with DMSO or DHODH inhibitors for 3 days. At this timepoint, the medium was replaced with compound free medium containing anti-CD3/CD28 and flow cytometry analysis was performed 4 days after activation. Representative experiment of 2 biological repeats. **C**, **D** T cell were activated with anti-CD3/CD28 and treated with brequinar with or without 50 μM uridine (Urd) for 72 h. The same experiment was performed in the absence of activation. In **C**, cells were stained with AnnexinV and propidium iodide. In **D** DNA synthesis was measured by EdU incorporation and DNA content was determined by propidium iodide staining. Bar graphs show the average values obtained with samples from three donors. Error bars correspond to standard deviation (SD) and p values were calculated by two-way ANOVA. **E** T cells from a healthy human donor were activated with anti-CD3/CD28 antibodies and treated with brequinar and uridine as indicated for 72 h. Viable cells were counted and equal numbers of viable T cells were used to test their ability to kill ^51^Cr labelled allogeneic LCLs. *n* = 3 technical repeats, error bars correspond to SD. **F** T cells were treated with DMSO or brequinar for 3 days. At this timepoint, the medium was replaced with compound free medium and cells were activated with anti-CD3/CD28 antibodies for 3 days. Viable cells were counted and equal numbers of viable T cells were used to test their ability to kill ^51^Cr labelled allogeneic LCLs. *n* = 3 technical repeats, error bars correspond to SD. **G** Inhibition of nucleoside uptake further decreases proliferation of brequinar-treated activated T cells. T cells were activated with anti-CD3/CD28 in the absence or presence of the indicated concentrations of brequinar and/or dipyridamole (DP). Cell growth was assessed by MTT assay after 4 days. *n* = 3 technical repeats, error bars correspond to SD. **H** T cells were activated as above in the absence or presence of the indicated concentrations of vehicle (DMSO) or brequinar and increasing concentrations of cytidine. *n* = 3 technical repeats, error bars correspond to SD and *p* values were calculated by Student’s *t*-test. For biological repetitions and additional information see Figures S2 and S5.

In a first set of experiments, we confirmed the antiproliferative effect of DHODHi on activated T cells isolated from healthy donors and cultured in the presence of inactivated fetal bovine serum (iFBS) (Fig. 1A). Resting T cells were insensitive to treatment with DHODHi and could proliferate upon removal of the inhibitor and subsequent activation (Fig. 1B). Pharmacologic DHODH inhibition on activated T cells caused an increase in cell death and a reduction in cell S-phase entry. As expected, these effects could be prevented through the salvage pathway by adding an excess of uridine to the culture medium (Fig. 1C, D). The cytotoxic function of T cells surviving the brequinar treatment was partially diminished (Fig. 1E and S2) even though the abundance of the CD69 early activation marker was not changed (Fig. S2). Resting T cells showed normal cytotoxic activity upon removal of the DHODHi and later activation (Fig. 1F and S2),

Activated T cells are known to be highly dependent on pyrimidine ribonucleotide synthesis and need to multiply these pools by 8-fold [15]. Reflecting this, the mRNA levels of pyrimidine nucleotide metabolism enzymes rapidly change in human T cells upon activation (Fig. S3). For example, after 4 h of activation, human T cells upregulate the mRNAs for the nucleoside transporter SLC29A1 and the uridine and cytidine kinase UCK2 [16]. Rises in the expression of some of these factors have also been detected at the protein level [17–19].

Uridine levels in human plasma are in the low μM range and fluctuate with during the day [20]. Because uptake of uridine by cells can hinder the effect of DHODH inhibition [21] uridine uptake blockage with small molecules such as dipyridamole (which blocks SLC29A1) has been suggested as a strategy to enhance the antitumor activity of DHODHi [22–24]. Here we investigated whether dipyridamole would also alter activated T cells. Whereas on its own dipyridamole had little effect, it enhanced the antiproliferative effect of brequinar on T cells (Fig. 1G). Therefore, if DHODHi are to be used together with nucleoside transport inhibitors in patients, the negative effects of this combination on T cell function must be considered.

Aside from uridine, mM concentrations of deoxycytidine were reported to rescue K562 leukemia cells from DHODH inhibition [25]. However, we failed to rescue activated T cells with this approach (Fig. S4). Adding thymidine, alone or with deoxycytidine did not have any effect on activated T cells either. Instead, we observed that addition of cytidine to the cell culture medium rescued T cells from DHODH inhibition (Fig. 1H). This suggests that activated T cells can use cytidine through the salvage pathway and that strategies to raise extracellular cytidine levels should rescue T cells from DHODHi.

### Solid cancers expressing low CDA levels

**Fig. 2:**
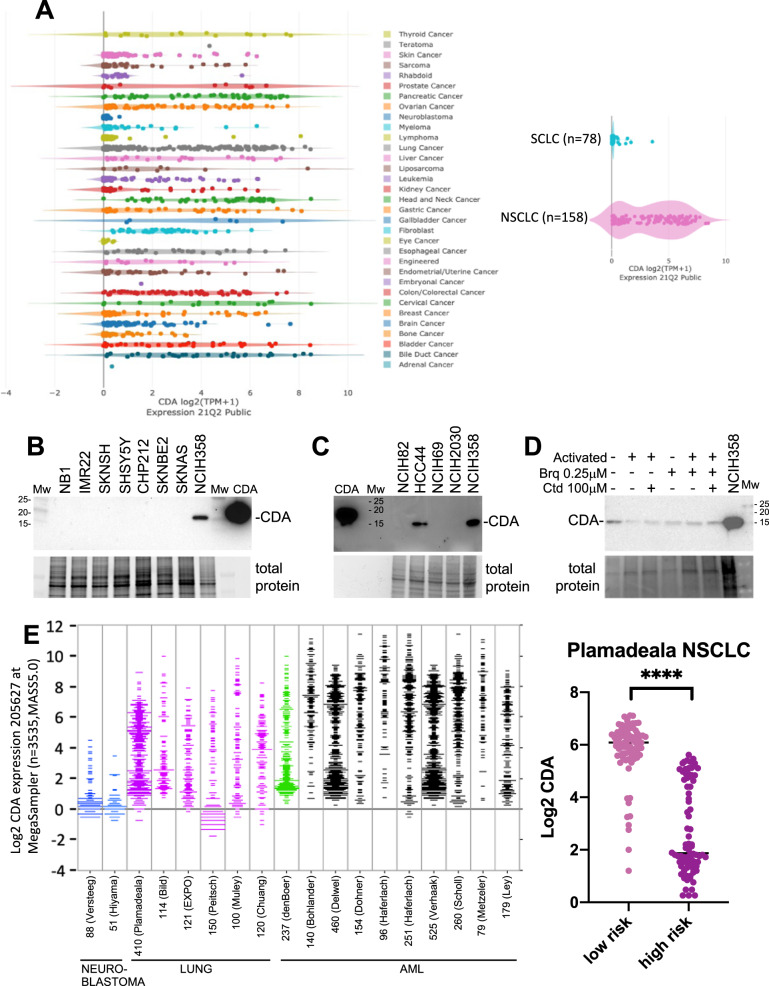
Identification of cancers with alterations in the expression of pyrimidine nucleotide metabolism enzymes. **A** Left: CDA mRNA expression levels in cell lines (Depmap portal). Right: CDA mRNA expression levels in SCLC and NSCLC cell lines (Depmap portal). **B**, **C** CDA protein expression analyzed by Western blots in neuroblastoma cell lines **B** and lung cancer cell lines **C**. See Figure S15 for full size blots. **D** CDA protein levels in non-activated and activated (48 h) T cells treated as indicated. See Figure S15 for full size blot. **E** Left: CDA expression levels in tumors (R2 Genomics Analysis and Visualization Platform). Number of samples in each study is indicated. Studies on neuroblastoma, lung cancer and AML containing more than 50 tumor samples are included. The denBoer data on childhood AML is highlighted in green. Right: Analysis of the Plamadeala data on NSCLC shows that high risk tumors have lower levels of CDA expression.

Searching for tumor types that could be hypersensitive to DHODH blockage, we compared the levels of mRNAs encoding proteins involved in the salvage pathway using the Depmap portal (https://depmap.org/portal/interactive/). This led to the finding that the mRNA levels of CDA are consistently low in several cell line types including cell lines derived from childhood cancers such as neuroblastoma (Fig. 2A). CDADC1 is another source of cytidine deaminase activity (Fig. S1) but there are no major differences in CDADC1 mRNA expression in cell lines from different tumor types except for chondrosarcomas (https://depmap.org/portal/interactive/). To support these observations, we studied the *CDA* gene expression in cell lines from the Cancer Cell Line Encyclopedia 21q4 (CCLE) database available in R2 (http://r2.amc.nl) and found a significantly lower expression of CDA (FDR < 0.01) in some pediatric cancers including neuroblastoma (two-sided Welch’s T-test, FDR <1 × 10^−35^, Table S1).

The genes encoding CDA and several other enzymes related with uracil and cytosine nucleotide metabolism locate to chromosome 1p (Table S2) and 1p deletions are frequent in neuroblastoma [26]. However, whether this influences CDA mRNA expression is unclear and all neuroblastoma cell lines tested show low CDA expression regardless of their 1p status (https://depmap.org/portal/interactive/). Although the average expression of CDA in the CCLE cell lines of neuroblastoma with 1p deletion was found to be lower (i.e. average log2[TPM] = 0.086) than in those without the rearrangement (i.e. average log2[TPM] = 0.170), this difference was not significant (Mann-Whitney U test, two-sided, *p-*value = 0.179, Table S3). In addition, the 1p region contains important genes for survival and proliferation aside from the genes involved in pyrimidine ribonucleotide metabolism (Fig. S1 and Table S2). All neuroblastoma cell lines tested were negative for CDA by Western blot analysis (Fig. 2B).

Most small cell lung carcinoma (SCLC) cell lines also express low levels of CDA mRNA (Fig. 2A) and as shown for NCIH69 and NCIH82 cells, this agrees with the absence of CDA protein detection (Fig. 2C and Table S2). Supporting these observations, we found a significantly lower expression of CDA (FDR < 0.01) for SCLCs from the CCLE (two-sided Welch’s T-test, FDR <1 × 10^−35^, Table S1). In this regard, it is interesting to point out that SCLCs are reported as highly sensitive to DHODH inhibition [9]. Non-small cell lung carcinoma (NSCLC) cell lines showed a significantly high expression of CDA in comparison to the other malignancies (two-sided Welch’s T-test, FDR = 3.18 × 10^−11^, Table S1). However, CDA expression levels are very variable (Fig. 2A). Cell lines such as NCIH2030 express low levels of CDA, whereas NCIH358 and HCC44 express higher levels (Fig. 2C and Table S4).

In addition, we checked for CDA levels in T cells. Although CDA mRNA is expressed in T cells at very low levels [16], CDA protein is detectable and at similar levels in resting and activated CD4+ and CD8 + T human cells [17]. Confirming this, in Fig. 2D, we show that CDA is detectable by Western blot in resting and activated T cells.

The CDA expression analysis on cancer cell lines were contrasted with data on tumor samples. Figure 2E shows data from the R2 Genomics Analysis and Visualization Platform for neuroblastoma, lung cancer and AML. According to this comparison, neuroblastoma tumor samples consistently show low levels of CDA expression (Mann-Whitney U test, two-sided, FDR = 0.006) while lung (most studies are on NSCLC) and AML show variable expression levels. For one of the lung cancer studies it was possible to associate high risk NSCLC with low CDA expression (Plamadeala study). It was also interesting to observe that childhood AML frequently show low CDA (denBoer study).

### Addition of an excess of cytidine sensitizes low-CDA expressing cancer cells to DHODHi when grown in medium supplemented with iFBS

Altogether, the observations described above suggested that cancer cell lines and T cells could respond differently to DHODHi, and in particular, to DHODHi in medium supplemented with cytidine.

**Fig. 3:**
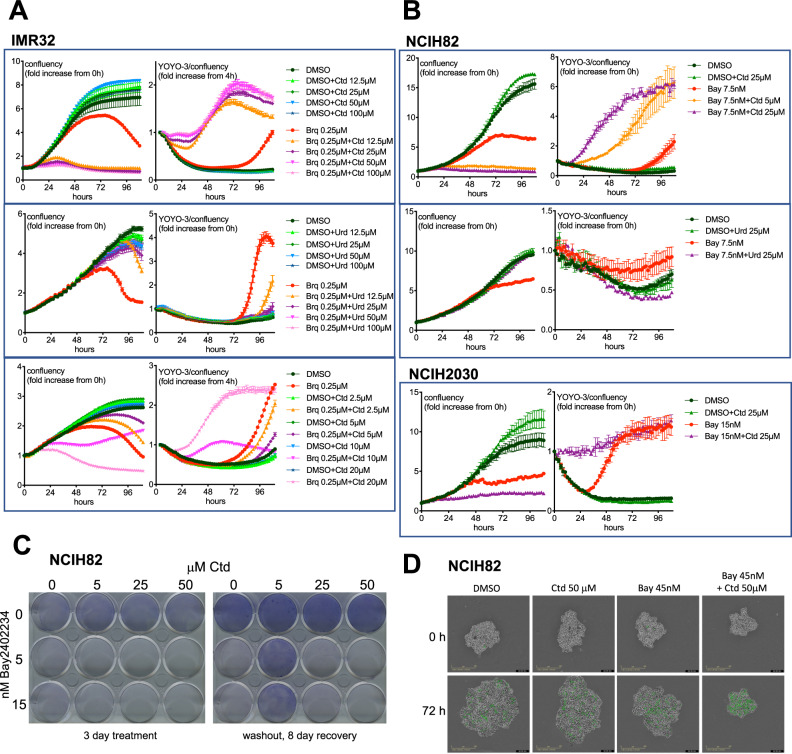
Supplementation with cytidine sensitizes low CDA expressing cells to DHODH inhibitors. **A**, **B** IMR32 (neuroblastoma), NCIH82 (SCLC) or NCIH2030 (NSCLC) cells were treated as indicated and cell growth (confluency) and death (YOYO-3 red/confluency) were measured by the IncuCyte method (See Materials and Methods). **C** Effect of the Bay2402234+cytidine combination on clonogenic assays with NCIH82 cells. **D** NCIH82 cells were grown as spheroids and treated as specified. Green colour indicates caspase3/7 activity.

As expected, addition of uridine to the cell culture medium protected IMR32 neuroblastoma cells from brequinar (Fig. 3A). In contrast and differing from the results obtained with activated T cells, cytidine did not protect IMR32 cells from DHODH blockage. Furthermore, addition of cytidine increased toxicity in the presence of brequinar and rapidly inhibited cell growth (Fig. 3A). To confirm that the response to the brequinar+cytidine combination is different for IMR32 cells and T cells, we performed experiments using the same seeding densities (Fig. S5). In Figs. S6 and S7 we tested other neuroblastoma cell lines. Of these, the ones where the brequinar+cytidine combination had the most dramatic and immediate effects were IMR32 and SHSY5Y. An increase in cell death was also evident in SKNSH and CHP212 cells. The weakest effects of the brequinar+cytidine combination were on SKNBE2 and SKNAS cells, both of which express mutant p53, a feature that is not prevalent in neuroblastoma. This may be of importance as DHODH inhibitors activate p53 [2]. NB1 cells were highly sensitive to brequinar on its own and cytidine did not enhance this sensitivity in our experimental conditions. Special properties of NB1 cells are that they are highly mutated in comparison with other neuroblastoma cell lines (963 mutations), they only have single *TP53* and *CDKN1A* copies, an extra copy of *CDADC1* and they are the only ones in the tested set with ALK amplification (https://depmap.org/portal/interactive). For a description of relevant alterations in the neuroblastoma cell lines tested here see Table S5.

### Intracellular CDA protects cells from the DHODHi + cytidine combination

To exclude that the toxicity of DHODHi+cytidine in cells grown with iFBS is only characteristic of neuroblastoma cells we compared its effects on lung cancer cell lines as these include low as well high CDA-expressing cells. Figure 3B, C shows enhanced toxicity in low-CDA expressing NCIH82 SCLC and NCIH2030 NSCLC cell lines treated with cytidine in combination with Bay2402234, another potent and specific DHODHi [8]. Even though both cell lines are deficient for p53 expression, in NCIH82 *RB1* is mutated and there is a 24-fold increase in c-Myc mRNA relative to normal cells (https://depmap.org/portal/interactive). NCIH2030 express oncogenic KRas, a feature reported to confer susceptibility to DHODHi [27, 28]. The DHODHi+cytidine combination also had a strong effect on NCIH82 cells when grown as spheroids (Fig. 3D).

**Fig. 4:**
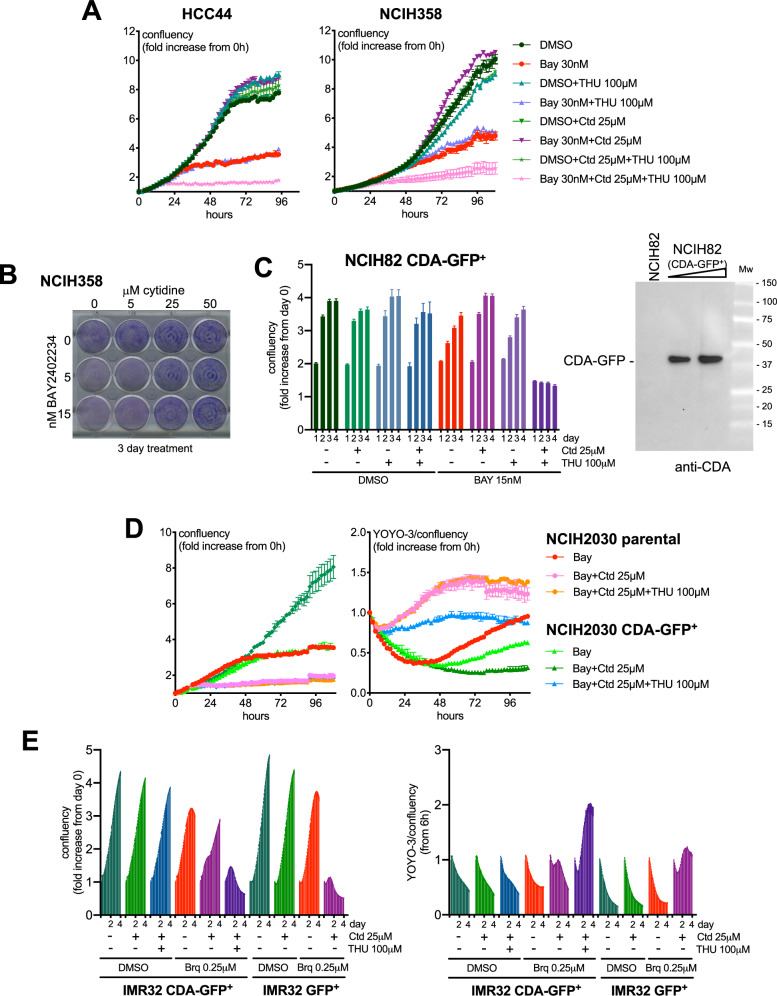
Intracellular CDA protects cells from the DHODHi+cytidine combination. **A** HCC44 or NCIH358 NSCLC cells were treated as indicated and analyzed by the IncuCyte method. **B** Effect of the Bay2402234+cytidine combination on clonogenic assays with NCIH358 cells. **C**–**E** CDA-GFP expression protects IMR32, NCIH2030 and NCIH82 cells from the DHODHi+cytidine combination. Cells were treated as indicated and cell growth (confluency) was measured by the IncuCyte method. A western blot demonstrating the expression of CDA-GFP is shown using an antibody against CDA.

In the case of two NSCLC cell lines that express high levels of CDA (HCC44 and NCIH358), cytidine effectively rescued from the DHODHi treatment (Fig. 4A, B). If the positive effect of cytidine on cancer cells expressing high-CDA and T cells is due to their CDA activity, it would be expected that the small molecule inhibitor of CDA tetrahydrouridine (THU) would prevent the rescue by cytidine of these cells from the DHODHi treatment. As shown in Fig. 4A and S8, this was the case. Accordingly, overexpression of CDA reduced the toxicity of the DHODHi+cytidine combination in the tested low-CDA cell lines (Fig. 4C–E). Human normal dermal fibroblasts were resistant to all treatments at least within a 4.5 day time frame (Figure S9).

One important issue in this study is that in some experiments, when used at the lower concentrations, cytidine could partly rescue cells from the DHODHi treatment also in low CDA expressing cells (e.g., Fig. 3A, C and S5A). This suggests that for the observations described here to be of therapeutic interest, sufficient extracellular concentrations of cytidine need to be achieved and sustained in vivo (see Discussion).

### Human serum allows low-CDA expressing cancer cells to proliferate in the presence of the DHODHi + cytidine combination

The results presented above were obtained with cells grown in standard cell culture conditions, that is in medium supplemented with inactivated fetal bovine serum (iFBS). To better resemble a physiologic setting, below we used medium supplemented with human serum (HS).

**Fig. 5:**
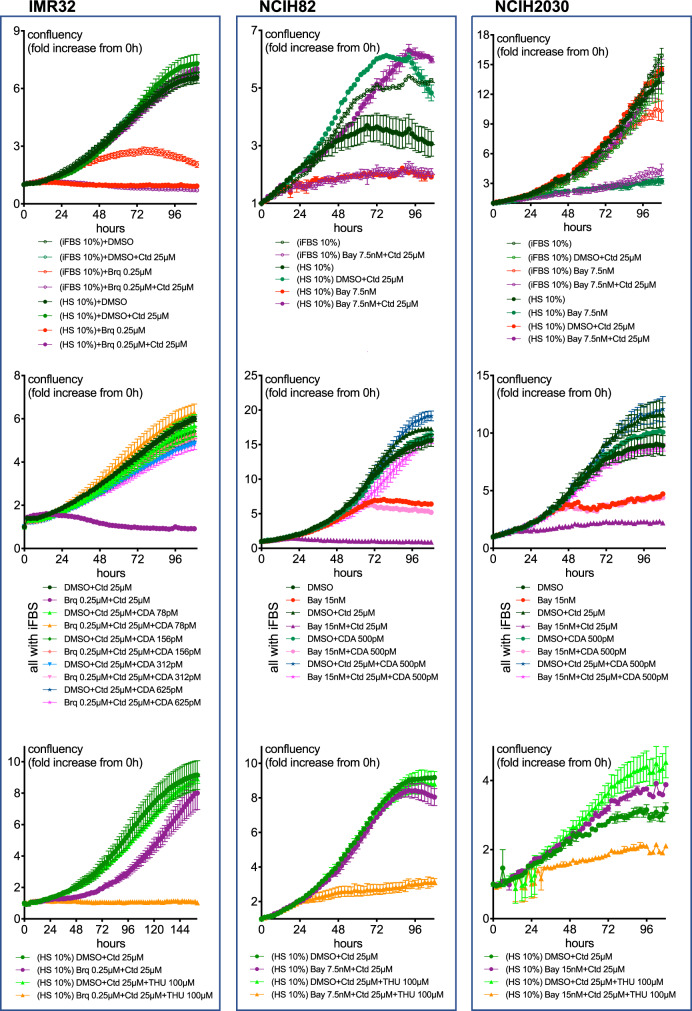
Human serum allows low-CDA expressing cancer cells to proliferate in the presence of the DHODHi+cytidine combination. IMR32, NCIH82 and NCIH2030 cells were grown in medium containing FBS or HS and the indicated compounds or recombinant human CDA. The effect of the treatment was measured by the IncuCyte system.

A comparison between Figs. 3 and 5 shows that IMR32, NCIH82 and NCIH2030 grown with HS were more sensitive to DHODHi on their own than when grown with iFBS. One possible way to explain this is that uridine levels are lower in HS than in FBS. Supporting this notion, enzymes involved in the degradation of uridine (DPYD, DPYS and UPB1) are present in human plasma (https://www.proteinatlas.org/). Accordingly, as exemplified in Fig. S10, IMR32 and NCIH82 cells grown with HS could be rescued by uridine from the DHODHi treatment. The observed hypersensitivity to DHODHi of cancer cells grown in HS is likely to be limited to cell culture experiments because in vivo, circulating uridine concentration is highly regulated and maintained in the 3–5 μM range [20].

Another clear difference between experiments performed with iFBS and HS was that low-CDA expressing cancer cells grown in HS were effectively rescued by the addition of 25 μM cytidine (Fig. 5). This was in marked contrast with results obtained in the presence of iFBS, where the DHODHi+25 μM cytidine combination is highly toxic to these low-CDA expressing cell lines (Fig. 3).

As mentioned above, one feature of human plasma/serum, is that it contains cytidine deaminase activity [29, 30] and CDA protein (https://www.proteinatlas.org/ and Fig. S11). Thus, we reasoned that the different response to the DHODHi+25 μM cytidine combination between cancer cells grown with HS and iFBS could be due to a more efficient conversion of cytidine into uridine in HS. Supporting this hypothesis, adding recombinant CDA to the cultures grown in iFBS rescued cells from the DHODHi+cytidine combination (Fig. 5). Further proof came from experiments performed with tetrahydrouridine showing that in the presence of this CDA inhibitor, IMR32, NCIH82 and NCIH2030 cells grown in HS could not proliferate upon treatment with the DHODHi+cytidine combination (Fig. 5).

Here it is interesting to note that cytidine at concentrations higher than 25 μM was less effective at protecting IMR32 cells grown with HS from brequinar (Fig. S12). This indicates that the CDA amount in HS (added at 10%) is not sufficient to protect these cells from the brequinar+cytidine when cytidine is added in a large excess. Accordingly, supplementing these cultures with recombinant CDA had a rescuing effect even at the highest concentration of added cytidine (Fig. S12).

All the experiments using FBS were performed with inactivated FBS. Instead, the experiments with HS were performed with non-inactivated serum. However, serum inactivation had no qualitative effect on the results (Fig. S13).

### Activated T cells treated with DHODHi + cytidine retain their cytotoxic activity

**Fig. 6:**
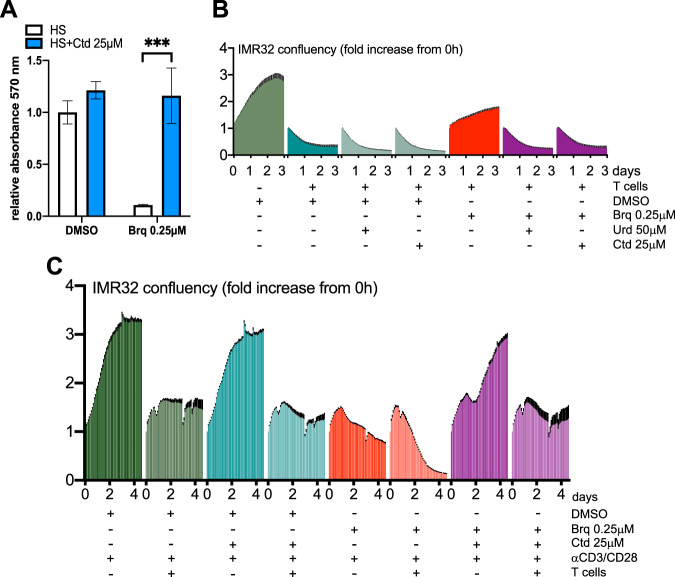
Activated T cells treated with DHODHi+cytidine retain their cytotoxic activity. **A** T cells were grown with HS and activated in the presence of the indicated compounds and cell growth was monitored by the MTT assay. Values correspond to the mean of three technical repeats, error bars correspond to standard deviation (SD) and *p* values were calculated by two-way ANOVA. **B** T cells were activated for 3 days in the presence of vehicle DMSO or brequinar with or without cytidine or uridine. All T cell treatments were performed in medium supplemented with HS. After the 3 days of activation, T cells were washed and 80,000 activated T cells were added to GFP labelled IMR32 cells cultured in compound free medium supplemented with HS. The presence of green cells was monitored with the IncuCyte system. All treatments were performed in triplicate. Error bars (SEM) are in black. **C** Co-culture experiment performed in medium supplemented with HS. 100,000 T cells were added to 40,000 GFP labelled IMR32 cells and where indicated, T cells were activated by adding anti-CD3/CD28 in the presence or absence of brequinar and/or cytidine. Cell growth of GFP labelled cells was monitored with the IncuCyte system. All treatments were performed in triplicate. Error bars (SEM) are in black.

As mentioned above, cancer cells grown with HS were very sensitive to DHODHi on their own. Hence, it could be interpreted that it is not necessary to combine DHODHi with other agents to eliminate tumor cells in humans. However, as mentioned above, this is unlikely to be reflected in vivo because plasma uridine levels are between 3 and 5 μM. Furthermore, the negative effect of brequinar on T cells grown with HS is also strong (Fig. 6A), so there was still a need to rescue the immune system from DHODH inhibition. Figure 6A shows that adding cytidine protected activated T cells grown with HS from brequinar as it did when T cells were grown with iFBS (Fig. 1H).

Next, we tested whether T cells treated with the DHODHi+cytidine combination not only proliferated but were also able to eliminate cancer cells. In a first experiment, we activated T cells in medium supplemented with HS with or without DHODHi+cytidine. After 3 days of activation, T cells were washed and a fixed number of viable T cells were added to GFP-labelled IMR32 cultures also grown with HS. As shown in Fig. 6B, activated T cells could eliminate IMR32 cells but their effect was weakened when they were activated in the presence of brequinar. Addition of uridine or cytidine during activation prevented this effect of brequinar.

The second challenge was to use a system where resting T cells are added to cancer cell cultures (in medium supplemented with HS) and then activated. As shown in Fig. 6C activated T cells could eliminate cancer cells in this co-culture system even in the presence of the brequinar+cytidine combination. However, not unexpectedly, cytidine had a positive effect on IMR32 cell confluency. Therefore, we needed to test conditions where the rescuing effect of cytidine on IMR32 cells is weakened (see below).

### Deoxycytidine sensitizes IMR32 cells grown with HS to the DHODHi + cytidine combination

**Fig. 7:**
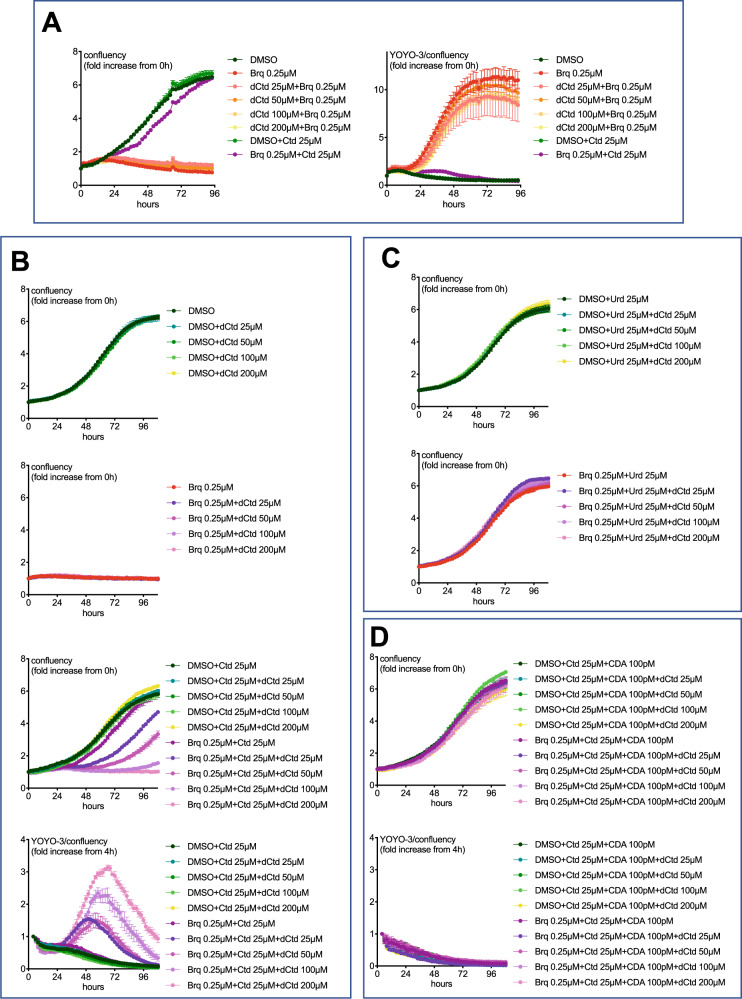
Effects of deoxycytidine on IMR32 neuroblastoma cells treated with brequinar or with the brequinar+cytidine combination in medium supplemented with HS. **A**–**D** IMR32 cells grown in medium supplemented with HS were treated with the indicated compounds or recombinant human CDA and their growth was monitored by the IncuCyte system. The data in panels **B**–**D** is from the same experiment.

It is known that deoxycytidine is a substrate for CDA [29] (confirmed in Fig. S14). Therefore, we tested whether like cytidine, deoxycytidine would protect IMR32 cells grown in HS from brequinar. However, unlike cytidine, deoxycytidine had no effect on either cell confluency or death induced by brequinar (Fig. 7A). From this observation we reasoned that deoxycytidine, by competing with cytidine for deamination by CDA, could abolish the rescuing effect of cytidine on IMR32 cells grown in HS and treated with brequinar. As shown in Fig. 7B, adding deoxycytidine achieved this. However, an alternative explanation for the effect of deoxycytidine is that it blocks the entry of uridine (uridine derived from the conversion of the added cytidine into uridine by the CDA in human serum). Disfavoring this possibility, Fig. 7C shows that deoxycytidine did not prevent the rescue of IMR32 cells by uridine. Furthermore, adding extra recombinant CDA to the culture medium precluded deoxycytidine from reducing the growth of IMR32 cells treated with brequinar+cytidine (Fig. 7D). Altogether, the results in Fig. 7 strongly support that adding deoxycytidine can compete with cytidine for binding to serum CDA.

### Deoxycytidine does not affect the ability of T cells to eliminate IMR32 cells

**Fig. 8:**
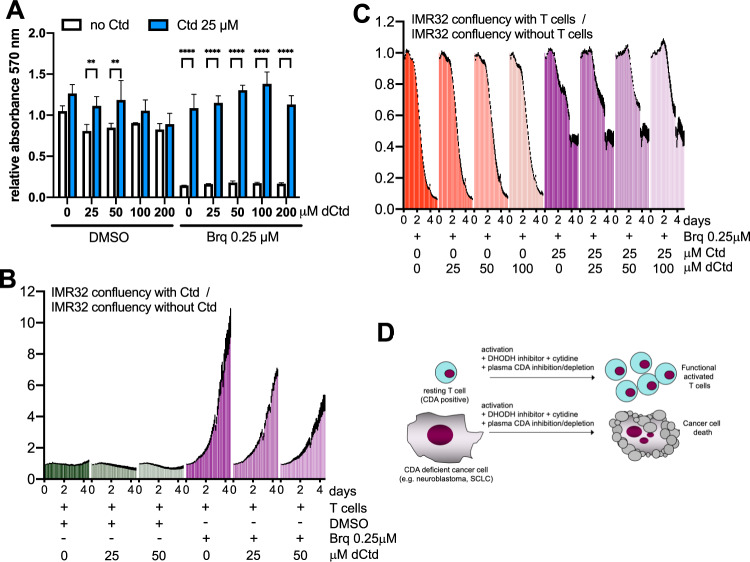
Deoxycytidine does not affect the ability of T cells to eliminate IMR32 cells. **A** T cells were grown with HS and activated in the presence of the indicated compounds and cell growth was monitored by the MTT assay after 4 days. Values correspond to the mean of three technical repeats, error bars correspond to standard deviation (SD) and *p* values were calculated by two-way ANOVA. **B** 100,000 T cells were added to 40,000 GFP-labelled IMR32 cells. Co-cultures were treated with anti-CD3/CD28 to activate T cells in the presence of the indicated compounds. Cell growth of GFP-labelled cells was monitored with the IncuCyte system and the ratio between the green cell confluency in the presence of 25 μM cytidine and the green cell confluency in the absence of cytidine was calculated. All treatments were performed in triplicate. Error bars (SEM) are in black. **C** The same experiment as above was carried out in the absence and in the presence of 100,000 T cells. All samples contained anti-CD3/CD28 and brequinar (0.25 μM). The ratio between the green cell confluency in the presence and the green cell confluency in the absence of T cells was calculated. All treatments were performed in triplicate. Error bars (SEM) are in black. **D** Working model.

As described above (Fig. S4) deoxycytidine does not protect activated T cells grown with iFBS from DHODH inhibition. Figure 8A shows that is also the case for T cells grown with HS. In addition, and in contrast with the results with IMR32 cells, deoxycytidine did not affect the rescue of T cells by cytidine.

Above we mentioned that according to the current literature, mouse plasma/serum contains significantly higher levels of cytidine than that of humans. In agreement with this data, and using the reagents available, we could not detect CDA in mouse serum (Fig. S11). Therefore, testing for the effect of decreasing plasma CDA in mouse models is not justified. As an alternative, we performed experiments using human cancer cells co-cultured with human T cells in medium containing human serum and assessed the effect of deoxycytidine. As in Fig. 7B, deoxycytidine reduced the rescue by cytidine of IMR32 cells also in these co-culture studies (Fig. 8B). More importantly, deoxycytidine did not prevent T cells from eliminating IMR32 cells in brequinar treated samples (Fig. 8C).

## Discussion

This study shows that extracellular cytidine protects activated T cells from DHODH inhibitors and therefore, that increasing cytidine levels in human plasma may reduce immunosuppression by these inhibitors. The evidence presented here also supports that low-CDA expression in cancers cells may boost susceptibility to DHODH inhibitors, especially when cytidine is in excess. As shown in the Fig. 2, cell lines from several cancer types consistently express low levels of CDA and there is a large proportion of cell lines from different cancer types with poor expression of this enzyme. These include most neuroblastoma cells lines and SCLC cell lines, and interestingly, promising results have been obtained in murine models for these diseases [6, 9]. Acute myeloid leukemias are not amongst the cancer types that consistently show low CDA levels, except perhaps for childhood AML according to the available data (Fig. 2E). However, it is in adult AML patients where cancer clinical trials have been and are being performed. Altogether, these observations indicate that increasing cytidine levels in plasma may provide a means to improve the therapeutic index of DHODHi especially in patients with cancers where CDA expression is low (see model in Fig. 8D).

One obvious question deriving from this work is whether it is possible to augment plasma cytidine to sufficiently high levels. Cytidine from diet is likely to be rapidly converted into uridine in the gastrointestinal tract and liver [31]. Therefore, cytidine needs to be injected or perhaps administered orally in the form of CDP-choline [32]. Unlike uridine, cytidine cannot be degraded by mammals (unless converted into uridine), which raises the chances of achieving high plasma concentrations. In mice, cytidine administered at 3500 mg/kg by i.p. bolus injection reached a peak concentration of about 10 mM after 1 h which was roughly maintained at least within 4 h. Cytidine was deaminated to uridine, but uridine levels did not exceed those of cytidine within the 4 h period [33]. In this regard, an important issue that must be considered when injecting pyrimidine nucleosides is that they may affect body temperature [20, 33–35]. However, at least in rabbits, unlike uridine injection, cytidine injection did not have an effect on body temperature [34].

Removing or blocking plasma CDA may help raising and maintaining plasma cytidine levels. Cell-permeable inhibitors such as THU may help to reduce plasma CDA activity and are in clinical trials in combination with approved cytidine analogues such as decitabine, which is inactivated by CDA [36, 37]. However, cell permeable inhibitors are bound to affect the intracellular CDA activity of normal cells including T cells and reduce their antitumor activity. Deoxycytidine, rather than blocking the enzyme like THU, may act by competing with cytidine for serum CDA and as shown here deoxycytidine has no deleterious effect on T cells. However, we view the results with deoxycytidine only as a proof-of-concept to justify the search for agents that can be used to block or remove (e.g., by plasmapheresis/immunoadsorption system [38]) plasma CDA. This is because it is most likely that in vivo, deoxycytidine will be taken up by cells or excreted and therefore rapidly disappear from circulation. Altogether, developing a non-cell permeable CDA inhibitor or a method to remove plasma CDA may be of immediate use in cancer patients not responding to cytidine analogues and, also perhaps to improve the therapeutic index of DHODH inhibitors.

Several reports advocate that plasma CDA derives from damaged neutrophils, which express high levels of CDA (https://www.proteinatlas.org/), are remarkably short-lived, infiltrate many tumor types and constitute 70% of WBC in humans and 20–30% of WBC in mice (this % increases with age) [36, 39–46]. This could indicate that CDA accumulates in plasma due to different types of stress over a lifetime. Indeed, circulating CDA was shown to be increased in several conditions including septicemia, metastasis and rheumatoid arthritis as well as in systemic lupus erythematosus patients [36, 39–42, 47, 48], and whereas uridine levels do not differ, plasma cytidine levels may be lower in adults than in infants [13]. Therefore, it is possible that infants or adult patients with low plasma/serum CDA, and that carry tumors defective for CDA expression, may respond better to DHODH inhibition and suffer from less side effects such as immunosuppression. These observations also indicate that reducing CDA activity in plasma is not likely to be deleterious. Altogether, targeting extracellular CDA, and perhaps other enzymes that accumulate in plasma may contribute to improve cancer treatment.

## Materials and methods

### Chemicals, proteins, and sera

Chemicals and their sources were as follows: Brequinar (Biotechne #6196; Sigma, SML0113), teriflunomide (Selleck S4169), Bay2402234 (Med Chem Express # HY-112645), uridine (Thermofisher scientific # A15227.14), cytidine (Sigma-Aldrich #C4654), tetrahydrouridine (Sigma-Aldrich #584223). Recombinant human CDA was from Sigma-Aldrich (#SRP6372). Inactivated fetal bovine serum was from Nordic biolabs (#SV30160.02) and human serum (whole blood) was from Sigma-Aldrich (#H6914).

### Separation of primary T cells

Peripheral blood mononuclear cells (PBMCs) from healthy donors were separated by Ficoll-Paque (GE Health Care #17-1440-02) density gradient separation. T cells from PBMCs were enriched, first by depleting B cells (Dynabeads CD19 Pan B (Invitrogen #11143D)), followed by monocyte depletion by plastic adhesion.

### T cell activation

To activate T cells, they were cultured in complete RPMI medium (containing 10% serum (iFBS or HS) and 1% penicillin/ streptomycin) supplemented with anti-CD3 (1.25 µg/mL, clone HTT3a, BioLegend #300314) and anti-CD28 (2 µg/mL, clone CD28.2, BioLegend #302923) antibodies.

### Estimation of number of cell divisions

Number of cell divisions were determined by following dilution of carboxyfluorescein succinimidyl ester, CFSE (AAT Bioquest #22028) stained T cells with a FACSCalibur (BD Biosciences) flow cytometer. T cells (10^6^ cells/mL in a 15 ml Falcon tube) were incubated with 5 µM CFSE in PBS with 5% iFBS for 5 min at room temperature. Subsequently, they were washed with PBS two times before subjecting them to activation and treatment with compounds for 48, 72, or 96 h in a 96 well format.

### Cell cycle analysis and Annexin V-PI flow cytometry

T cells were seeded at 1 × 10^5^ cells per well of a 96-well plate and left untreated (resting) or activated. Cell cycle distribution analysis was performed on a FACSCalibur (BD Biosciences) flow cytometer after labelling with 5-Ethynyl-2′-deoxyuridine (EdU) and staining with propidium iodide (Invitrogen #P3566). EdU labelling was performed according to the manufacturer’s protocol of the Click-iT™ EdU Alexa Fluor™ 488 Flow Cytometry Assay Kit (ThermoFisher, Scientific #C10425). Briefly, cells were pulsed with 10 µM EdU for 45 min and subsequently fixed with the reagents provided. Before analysing cells by flow cytometry, propidium iodide at 20 µg/mL and RNAse (Invitrogen #12091) at 100 µg/mL concentration were added to the cells. For the detection of apoptotic cells, the Annexin V-FITC Apoptosis detection kit (Abcam #ab14085) was used. Analysis conducted with the FlowJo software V 10.2.

### T cell cytotoxicity assay

Isolated T cells were treated and activated as indicated in the figure legends and cell viability was determined by trypan blue staining. For each cytotoxicity experiment the same number of live T cells was used. 1 × 10^4 51^Cr-labeled allogeneic lymphoblastoid cell lines (LCLs) cells were cultured with serially diluted activated T cells in triplicate for 4 h at 37 °C in 5% CO_2_. Radioactivity in the supernatant of cultures was measured with a gamma counter (Perkin Elmer).

### Cell proliferation measurements by MTT assay

A total of 1 × 10^5^ T cells were seeded on day 0 in 96-well plates in 200 µL of complete RPMI medium. Cells were activated by adding anti-CD3/CD28 and treated as described. At the indicated time points, plates were spun and supernatant removed. 100 µL of MTT solution (3 mg/mL of MTT (Thiazolyl Blue Tetrazolium Bromide, Sigma #M2128) dissolved in PBS: phenol red free RPMI (1:5)) were added to the cells and samples were incubated for 3 h at 37 °C in 5% CO_2_. Plates were spun again, the supernatant was discarded and plates were frozen at −20 °C. Plates were thawed at room temperature and MTT crystals were dissolved in 25 µL Sorensen’s Glycine buffer and 200 µL DMSO. Plates were kept shaking in dark for 10 min and absorbance was measured at 570 nm using a microplate reader (Tecan).

### Surface and intracellular marker analysis

A total of 1 × 10^5^ T cells per well of a 96-well plate were seeded and treated as indicated. To detect surface markers, T cells were first stained with Fc block to prevent unspecific binding (Invitrogen #L34970) and fixable aqua live dead staining (BD Pharmingen #564220) for 10 min at room temperature in the dark. After washing, cells were incubated with antibodies to CD4 (PE-Cy7, clone OKT4, BioLegend #317414), CD8 (APC, clone HTT8a, BioLegend #300912) and CD69 (FITC, clone FN50, BioLegend #310904) for 30 min in the dark at 4 °C. Intracellular staining for IFN-γ (BV 421, clone 4 S. B3, Biolegend #502532) and granzyme B (Alexa Fluor 647, clone GB11, Biolegend #515406) was performed using FoxP3/Transcription Factor Staining Buffer Set (eBioscience #00-5523-00) according to the manufacturer’s instructions. Stained cells were detected on a BD LSR-II flow cytometer. All flow cytometry data analysis was performed using FlowJo software V10.2.

### Cell lines

Neuroblastoma cell lines: IMR32, SKNBE2, SKNAS, SKNSH and SHSY5Y were a kind gift from Marie Arsenian Henriksson’s lab. CHP212 and NB1 were a kind gift from Susanne Schlisio’s lab.

SCLC cell lines (NCIH82, NCIH69) and NSCLC cell lines (NCIH2030, NCIH358 and HCC44) were purchased from the ATCC. Except for the spheroid experiment in Fig. 3D, NCIH82 cells were adapted to grow as monolayers. Single tandem repeat analysis conducted by Public Health England verified the integrity of the NCIH82 cells adapted to grow as monolayers. Profile match was 96%. Human normal dermal fibroblasts (HNDF) were purchased from PromoCell.

IMR32 cells were grown in Nutrient mixture F12 (Sigma-Aldrich #N6658) + Minimum Essential Medium Eagle (Sigma-Aldrich #M4655) (1:1), FBS 10% (Nordic biolabs #SV30160.03HI), Pen-Strep 1% and Non-essential amino acids 1%. SKNBE2, SKNAS, SKNSH and SHSY5Y were grown in 50% DMEM (high glucose) and 50% F12 medium from Sigma-Aldrich supplemented with 10% FBS and 1% penicillin/streptomycin. CHP212 and NB1 were grown in DMEM medium from Sigma-Aldrich (high glucose) supplemented with 10% FBS and 1% penicillin/streptomycin. NCIH82, NCIH69, NCIH2030, NCIH358 and HCC44 were grown in RPMI medium (Sigma-Aldrich #R8758) supplemented with 10% iFBS and 1% penicillin/streptomycin. All cells were grown at 37 °C and 5% CO_2_.

### Western blotting

Culture medium was removed and cells were washed twice with 1 × PBS. Samples were lysed with 150 µL of 1 × LDS sample buffer (106 mM Tris-HCl (Sigma-Aldrich #T5941), 141 mM Trisma base (Sigma-Aldrich #1503), pH 8.5, 2% Lithium Dodecyl Sulphate (LDS), (Sigma-Aldrich #L9781), 10% glycerol (Sigma-Aldrich #G5516), 0.51 mM EDTA (Sigma-Aldrich #ED)), heated at 95 °C for 5 min, and sonicated for 3 × 10 s. After brief centrifugation at 16,000 × g, protein concentrations were determined with the Bio-Rad DC Protein Assay kit (BioRad #50-0116). Protein concentration was normalized between samples and 4 × Laemmli buffer (BioRad #161-0747) was added to a final concentration of 1×. DTT (Sigma-Aldrich #43819), used as reducing agent, was added to a final concentration of 100 mM and samples were heated at 95 °C for 5 min. Equivalent amounts of total protein were loaded on 12-well stain free 4–15% TGX gels (BioRad #456-8085) in standard tris-glycine running buffer (BioRad #161-0732) and the electrophoresis was run at 150 V. Gels were activated for 5 min using the ChemiDoc Touch (BioRad #170–8370) stain free gel activation protocol. Subsequently, proteins were transferred on a PVDF membrane (Trans-Blot Turbo kit, BioRad #170–4150) using the standard semidry transfer of BioRad Trans-Blot Turbo transfer system pre-set program (30 min, 25 V, 1 A).

Membranes were blocked with 5% milk in PBS-T (1 × PBS, 0.1% Tween 20 (Sigma-Aldrich #P9416)). Incubation with primary antibodies was carried out overnight at 4 °C under gentle agitation. Incubation with secondary antibody (Rabbit Anti Mouse-HRP (DAKO #P0261) was for 1 h on a shaker. Images were acquired using a ChemiDoc Touch system for both the chemiluminescence mode after development using Clarity Western ECL Substrate (BioRad #170–5061) or stain free membrane mode for the total protein loading. The primary antibody against CDA was SCBT #sc-365292 and used at 1 μg/mL.

### CDA ELISA and activity assay

The human CDA ELISA kit was from Cusabio (# CSB-EL004976HU-96), the mouse CDA ELISA kit was from Aviva (#OKEH04970-96) and the CDA activity assay kit was from BioVision (#K451).

### Overexpression

A plasmid expressing CDA with a GFP tag was purchased from Origene (#RG208922). IMR32 cells were transfected by using lipofectamine3000 from Thermofisher (#L3000001) according to instructions by the manufacturer. CDA-GFP expressing cells were enriched by G418 resistance. NCIH2030 cells were transfected in the same way, and cells overexpressing CDA-GFP were enriched by G418 resistance followed by GFP-positive cell sorting using the BD FACSAriaFusion.

### Live cell imaging analysis

Cells were seeded in a 96-well plate at a subconfluent density in 100 μL of medium with the indicated compounds. Unless otherwise specified, seeding densities were 5,000 cells/well (IMR32, SKNSH, SHSY5S, SKNBE2, SKNAS, NB1, CHP212 and HCT116), 3000–4000 cells/well (NCIH358), 2000 cells/well (NCIH82 and U2OS), 1500 cells/well (HNDF) and 1000 cells/well (NCIH2030 and HCC44). Next day, cultures were supplemented with 0.3 μM YOYO-3 (Invitrogen #Y3606). Plates were placed in an IncuCyte S3 system located inside a CO_2_ incubator for the times indicated with images taken every 2 h. Confluency % and the number of YOYO-3 positive cells (dead cells) were determined with the IncuCyte S3 2018A Rev 1 software. Confluency % was estimated as the percent of the image area occupied by objects. The number of YOYO-3 positive cells in each well was divided by the cell confluency % in the well. Data was plotted with GraphPad Prism v.8 displaying the mean value of at least three technical repeats ± SEM between three replicates.

### Spheroid experiments

NCIH82 cells grown as suspension cultures were collected by centrifugation at 1200 × *g* for 5 min. The supernatant was discarded, and the cell pellet was resuspended in 1 mL of complete RPMI medium (supplemented with iFBS). Viable cells were counted and cell suspension volume was adjusted to 12,500 viable cells/ml. 80 μl of this cell suspension added to each well of an ultra-low attachment 96-well plate (Costar #7007). Cells were observed under the microscope. If single cells were not clumped in a major cluster, the plate was tapped to promote aggregation. Cells were incubated for 2 days at 37 °C. On day 2, 100 μl of complete RPMI medium was carefully added to each well. On day 5 spheroids were clearly formed and the old medium was removed and replaced by 100 μl of fresh complete medium and indicated compounds were added to a final volume of 200 μl. Samples were supplemented with 0.375 μM caspase 3/7 reagent (Invitrogen #C10423). Plates were placed in an IncuCyte S3 system located inside a CO_2_ incubator with images taken every 4 h after an initial incubation period of 30 min.

### Statistical analyses

Sample sizes were *n* = 3–4 technical repeats as specified in each figure legend. Statistical methods are described in each figure legend and were performed using Graphpad Prism version 8.4.3. *P* values: * < 0.05, ** < 0.01, *** < 0.001, **** < 0.0001.

mRNA expression in 1389 cell lines from the CCLE Cancer Cell Line Encyclopedia (21q4) was obtained from the R2 database (https://hgserver1.amc.nl/cgi-bin/r2/main.cgi) using datagrabber. Similarly, the microarray expression of various tumors measured as MAS5.0 were obtained using the MegaSampler tool in R2. Welch’s T- and Mann-Whitney tests were conducted with the scipy package using the hypothesis described in the manuscript. Multiple test corrections were conducted using the Benjamini-Hochberg approach using the statsmodel package.

## Supplementary information

Supplementary figures and tables.

Figure S15: original data

## Data Availability

The data used to support the findings of this study are available from the corresponding authors upon request.
