# Correction: Proof-of-principle studies on a strategy to enhance nucleotide imbalance specifically in cancer cells

**DOI:** 10.1038/s41420-022-01275-z

**Published:** 2022-11-30

**Authors:** Twana Alkasalias, Juan Zhang, Harsha Madapura, Basile Dalarun, Oscar Bedoya Reina, Rolf Lewensohn, Kristina Viktorsson, Abbas Salihi, Suhas Darekar, Sonia Laín

**Affiliations:** 1 Department of Microbiology, Tumor and Cell Biology (MTC), Biomedicum, Karolinska Institutet, Stockholm, Sweden; 2 General Directorate of Scientific Research Center, Salahaddin University-Erbil, Erbil, Kurdistan Region Iraq; 3 Science for Life Laboratory, Stockholm, Sweden; 4 Department of Oncology-Pathology, BioClinicum, Karolinska Institutet, Stockholm, Sweden; 5 Theme Cancer, Patient Area Head and Neck, Lung and Skin, Karolinska University Hospital, Stockholm, Sweden; 6 Department of Biology, College of Science, Salahaddin University‑Erbil, Erbil, Kurdistan Region Iraq

**Keywords:** Targeted therapies, Cancer metabolism

Correction to: *Cell Death Discovery* 10.1038/s41420-022-01254-4, published online 24 November 2022

The original version of this article contained an error in an author name. Basile Dalaroun needs to be corrected to Basile Dalarun. The original article has been corrected.

