# Supplementary material for: Proof-of-principle studies on a strategy to enhance nucleotide imbalance specifically in cancer cells
Source: Cell Death Discov. 2022 Nov 24;8:464. doi: 10.1038/s41420-022-01254-4 (PMC9691752; doi:10.1038/s41420-022-01254-4)
Supplement: Supplementary file 1 — Supplementary figures and tables. [file 41420_2022_1254_MOESM1_ESM.pdf]

Proof-of-principle studies on a strategy to enhance nucleotide imbalance specifically in cancer cells

Alkasalias, Zhang et al., manuscript

Supplemental Figures and Tables

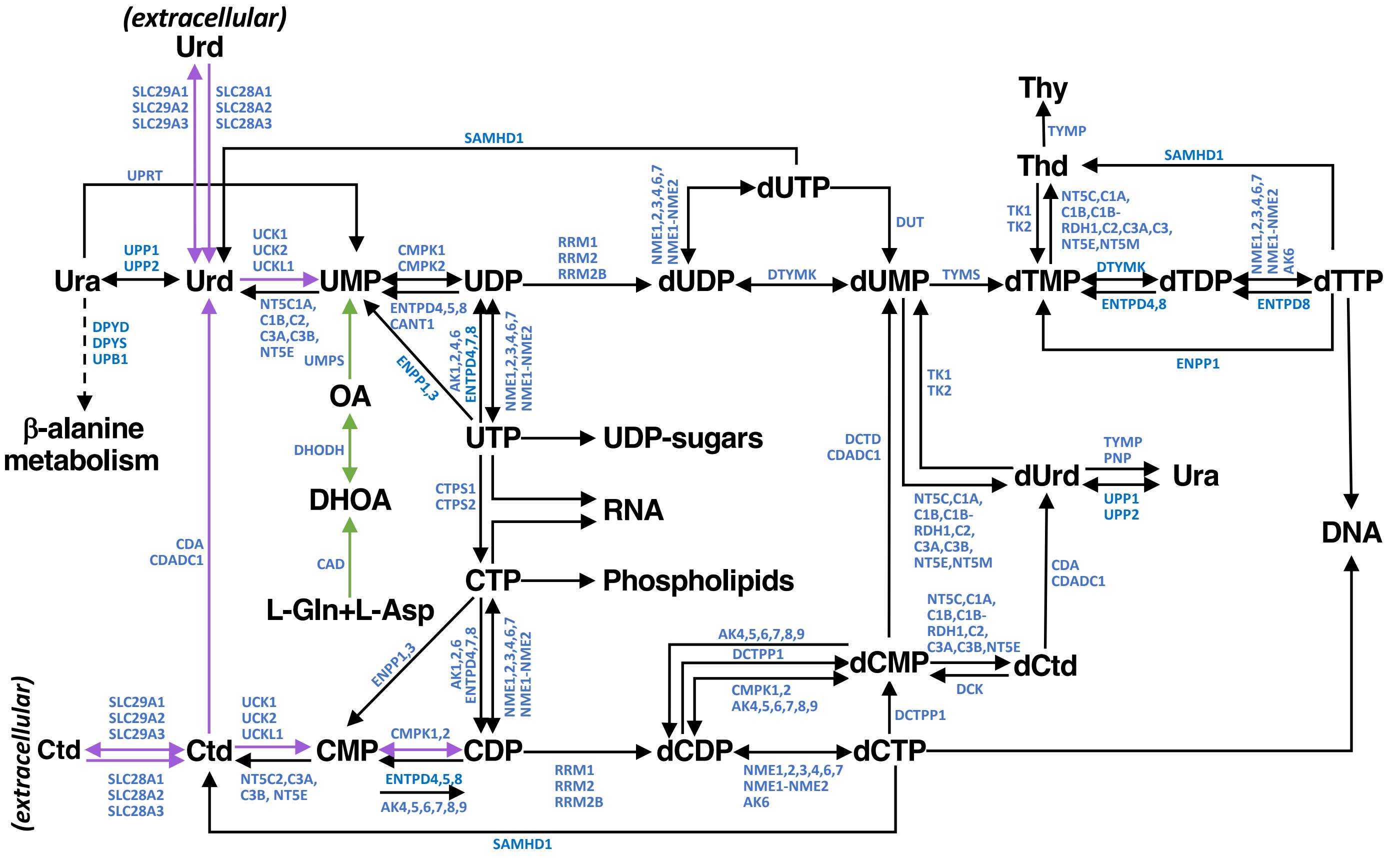

Figure S1. Pyrimidine nucleotide metabolism according to KEGG, GeneCard and additional literature [38,39]. Green and purple arrows correspond to the *de novo* and salvage pathways, respectively.

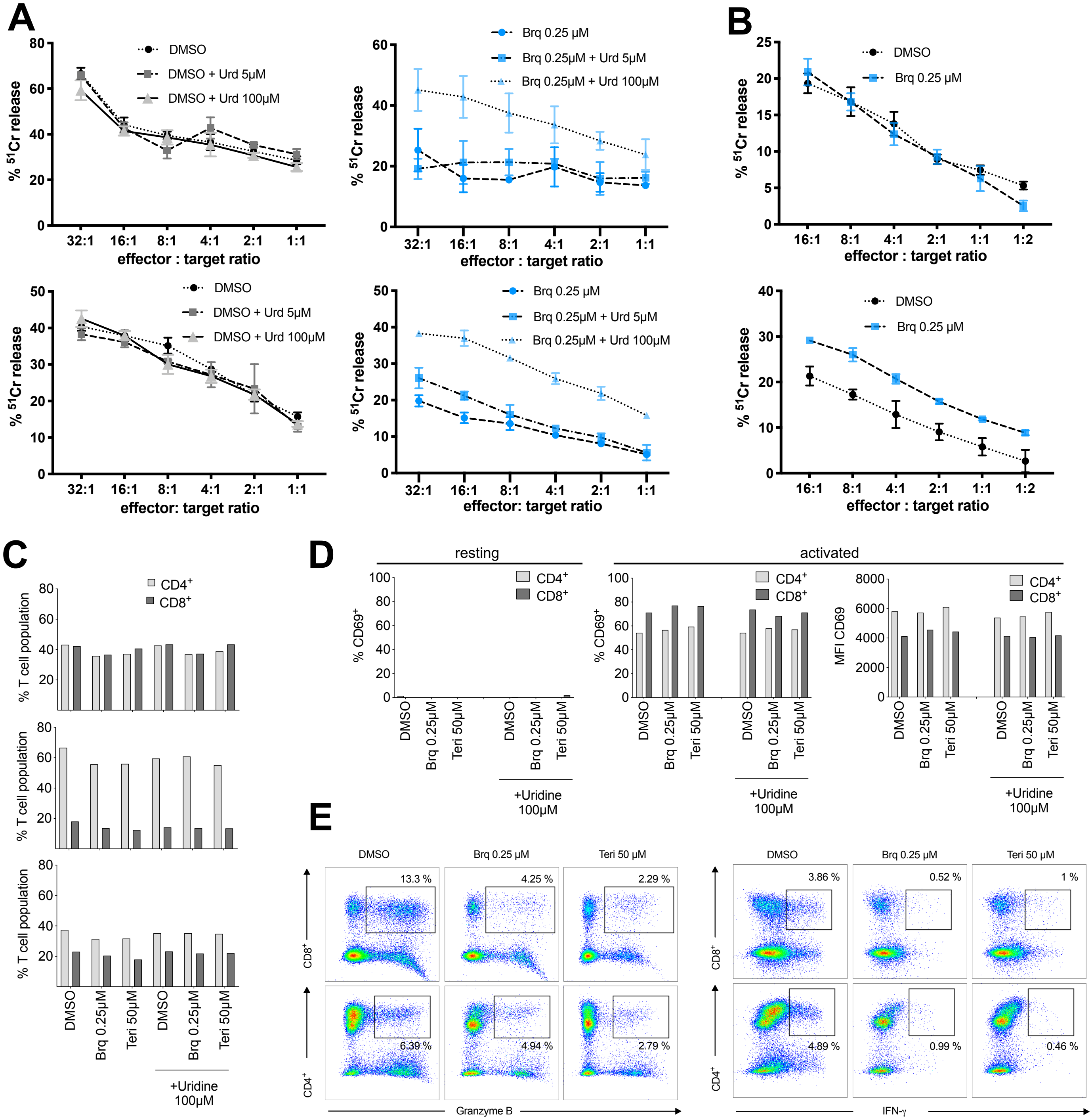

Figure S2. Effect of DHODH inhibitors on T cells. (A) T cells from two healthy human donors were activated with anti-CD3/CD28 and treated with brequinar and/or uridine for 72 h. Equal numbers of viable T cells were used to test their ability to kill ^{51}Cr labelled allogeneic LCLs. Chromium release values correspond to the average of three technical repeats ± SD. (B) T cells from two healthy donors were treated with DMSO or DHODH inhibitors for 3 days. At this timepoint, the medium was replaced with compound free medium and cells were activated with anti-CD3/CD28 antibodies for 72 h. Chromium release was performed as above. (C) T cells from three donors were activated by anti-CD3/CD28 antibodies for 72 h in presence of DMSO or DHODH inhibitors. The % of $CD4^+$ and $CD8^+$ T cells was determined by flow cytometry. (D) CD4+ or CD8+ T cells from a healthy donor were cultured in the presence of DMSO or DHODH inhibitors without mitogen (resting) or with anti-CD3/CD28 (activated) for 48 h after which the % of CD69 positive cells and the median fluorescence intensity (MFI) were analyzed by flow cytometry. (E) T cells were activated with anti-CD3/CD28 antibodies together with DHODH inhibitors for 96 h after which they were stained for granzyme B and interferon gamma (IFN-γ) and analysed by flow cytometry.

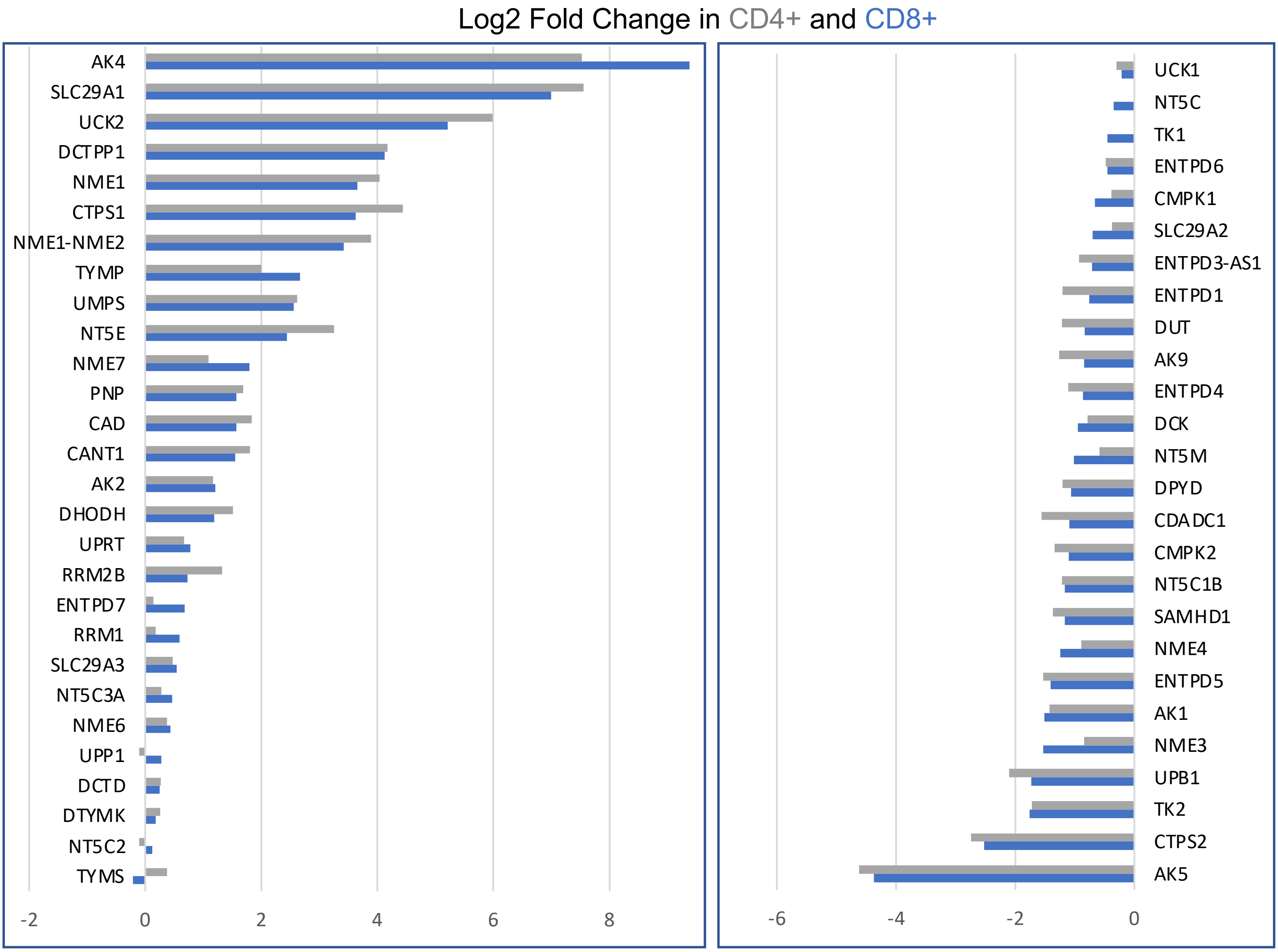

Figure S3. Effect of a 4 h activation on the expression of mRNAs encoding enzymes involved in pyrimidine nucleotide metabolism in CD4+ or CD8+ T cells according to Schmiedel et al., 2018.

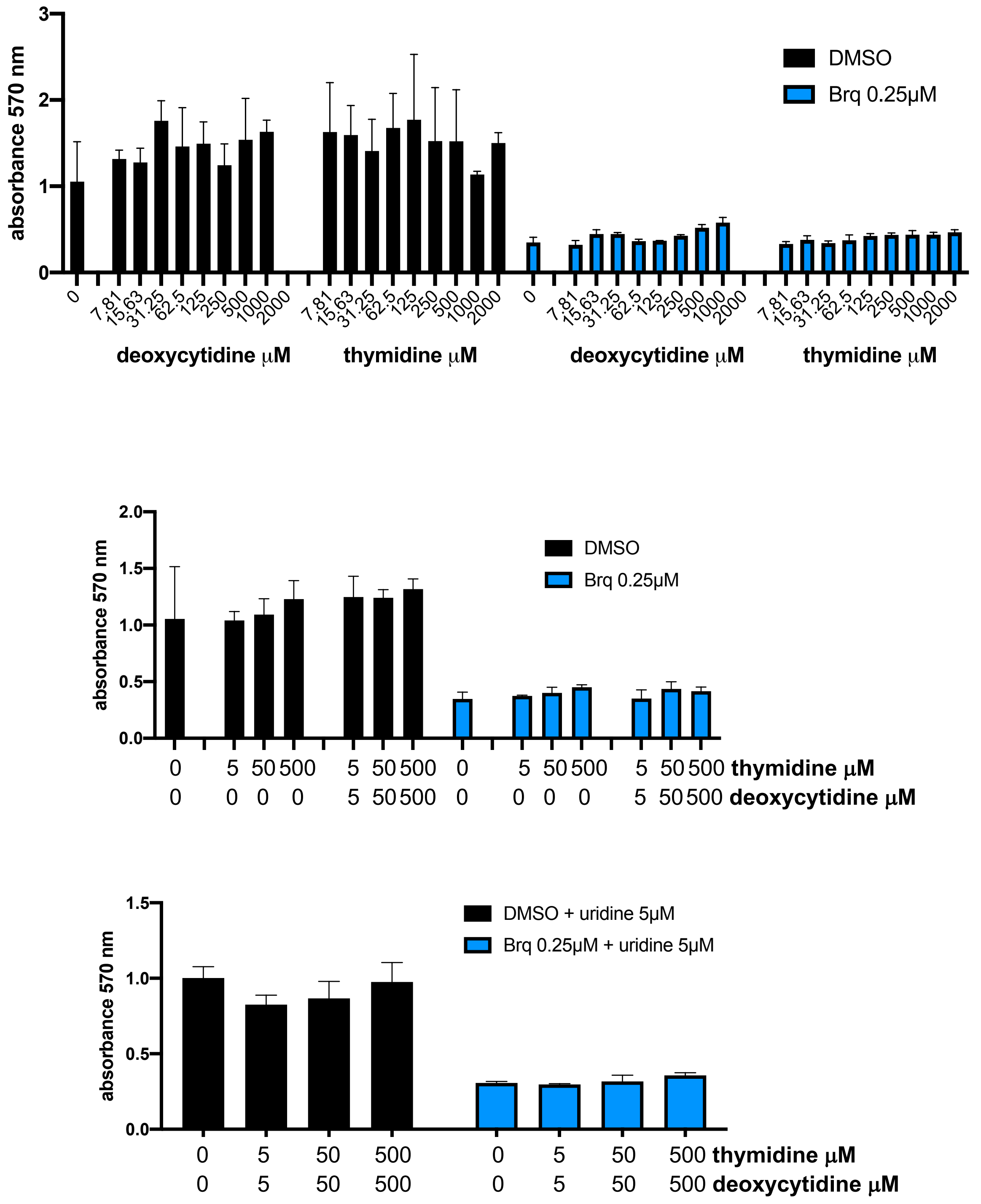

Figure S4. Deoxycytidine and thymidine do not protect activated T cells from DHODH inhibition. T cells were activated with antiCD3/antiCD28 in the absence or presence of the indicated concentrations of brequinar, deoxycytidine and/or thymidine. Cell growth was assessed by MTT assay. n=3 technical repeats, error bars correspond to SD.

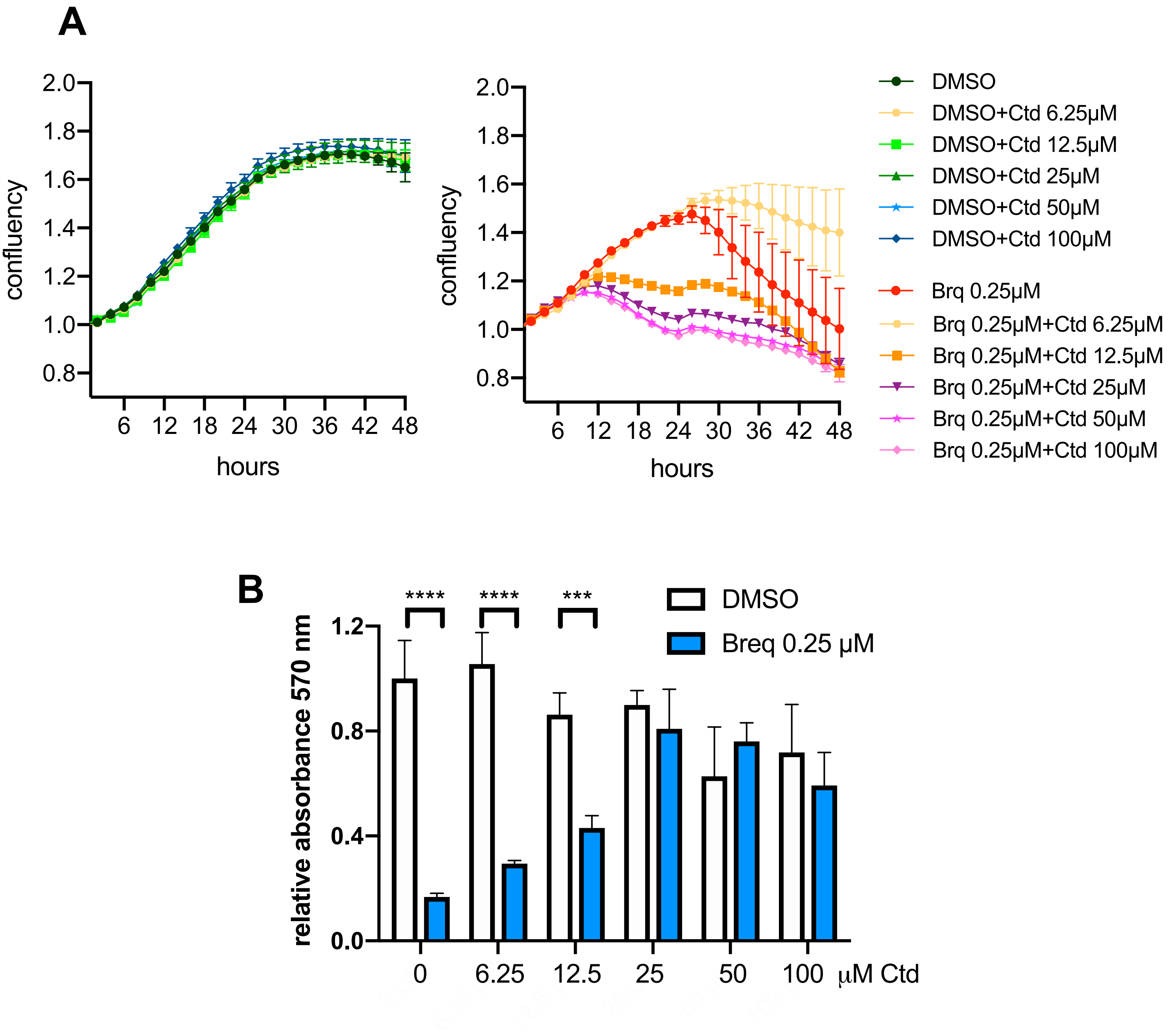

Figure S5. Cytidine sensitizes IMR32 neuroblastoma cells and protects activated T cells to brequinar when grown in the same conditions.

(A) IMR32 cells were seeded into 96 well plates (20,000 cells/well) and cell confluency were measured with the IncuCyte system. Error bars indicate SEM and n=4 technical repeats.

(B) T cells were seeded into 96 well plates (20,000 cells/well) and activated with antiCD3/antiCD28 for 5 days in the absence or presence of the indicated concentrations of brequinar and cytidine. Cell confluency was assessed by MTT assay. n=3 technical repeats, error bars=SD, two-way Anova.

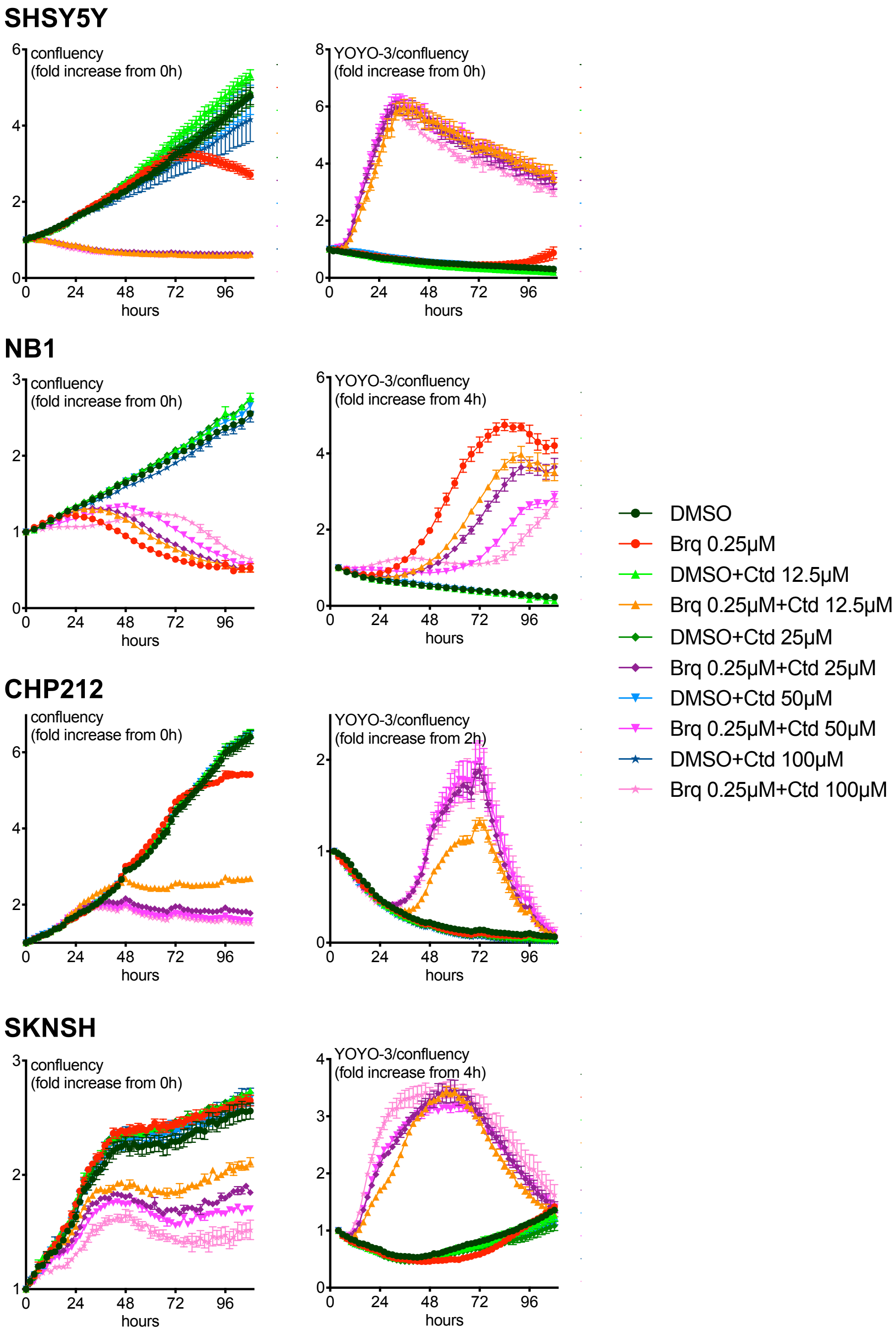

Figure S6. Effect of the brequinar + cytidine combination on neuroblastoma cells grown in the presence of iFBS. Cell confluency as well as the proportion of YOYO-3 positive cells were measured with the IncuCyte system.

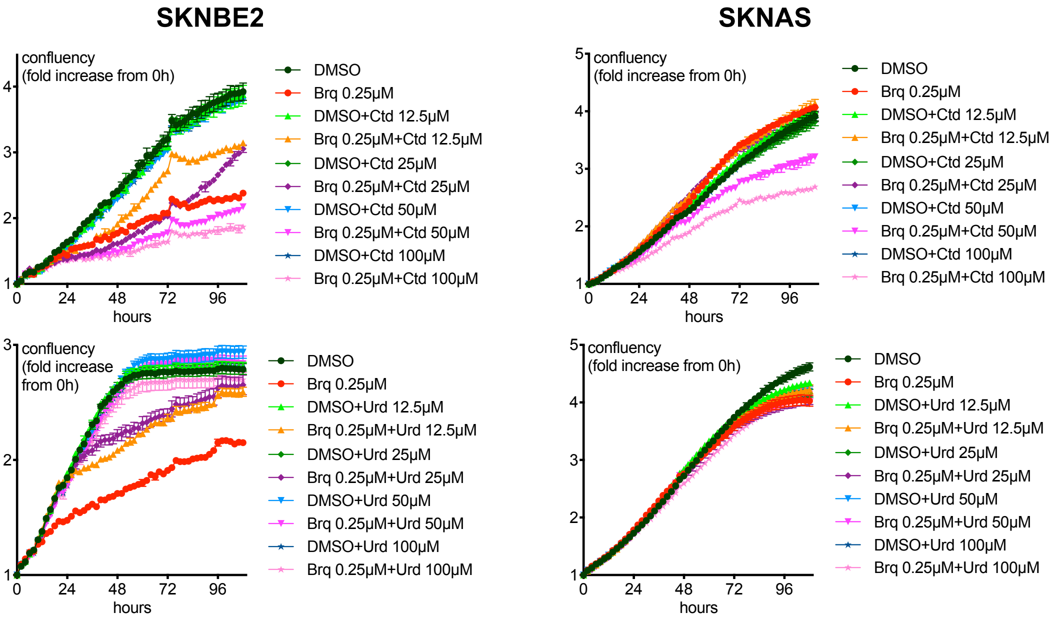

Figure S7. Effect of cytidine on DHODH inhibitor treated SKNBE2 and SKNAS p53-defective cells. Cells were treated with the indicated compounds over time and cell confluency was measured with the IncuCyte system.

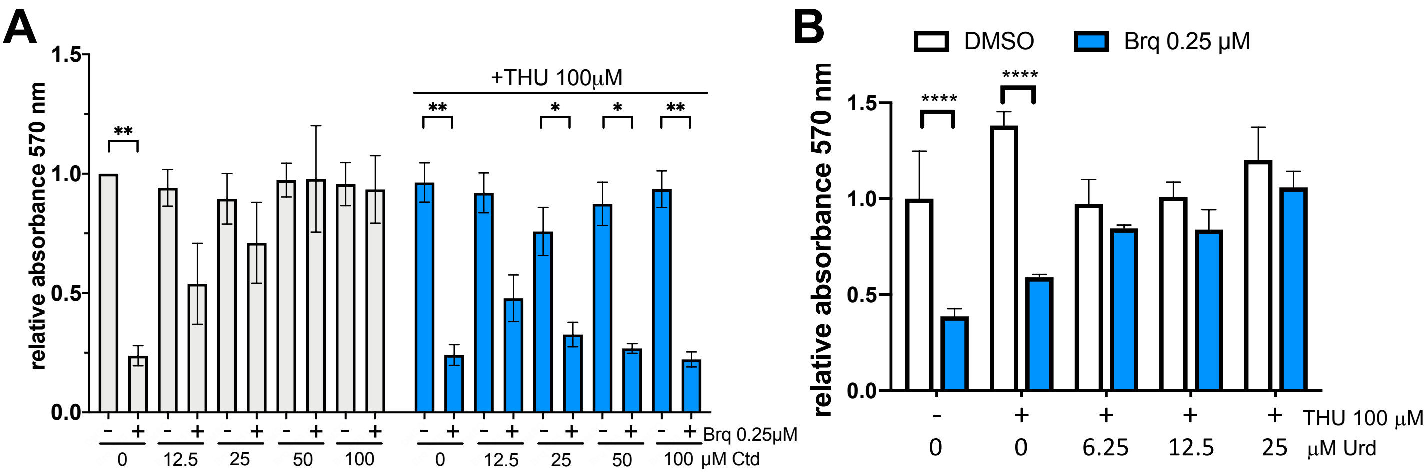

Figure S8. THU impairs the proliferation of T cells treated with the brequinar + cytidine combination. (A) Effect of THU on activated T cells grown in iFBS and treated with brequinar + cytidine (n=4 biological, repeats, error bar=SD, paired t test). (B) THU does not impair rescue by uridine suggesting that THU is acting in a specific manner against CDA (n=3 technical repeats, error bar=SD, two-way Anova) .

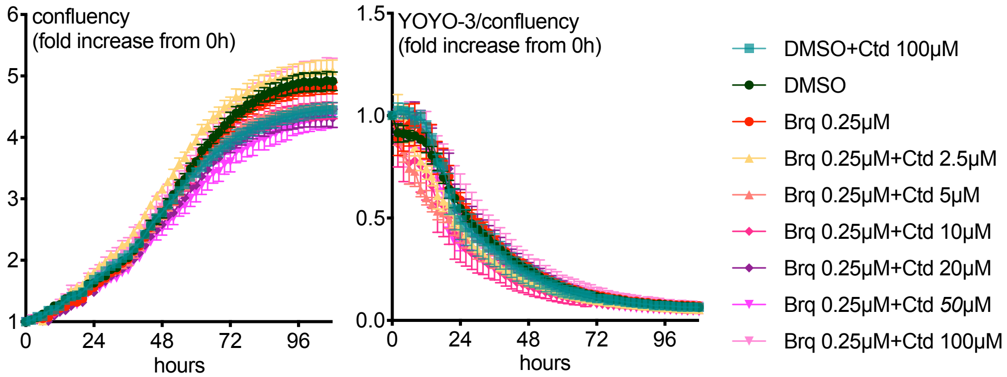

Figure S9. Effects of DHODHi + cytidine on human normal dermal fibroblasts (HNDF). Cell confluency as well as the proportion of YOYO-3 positive cells were measured with the IncuCyte system.

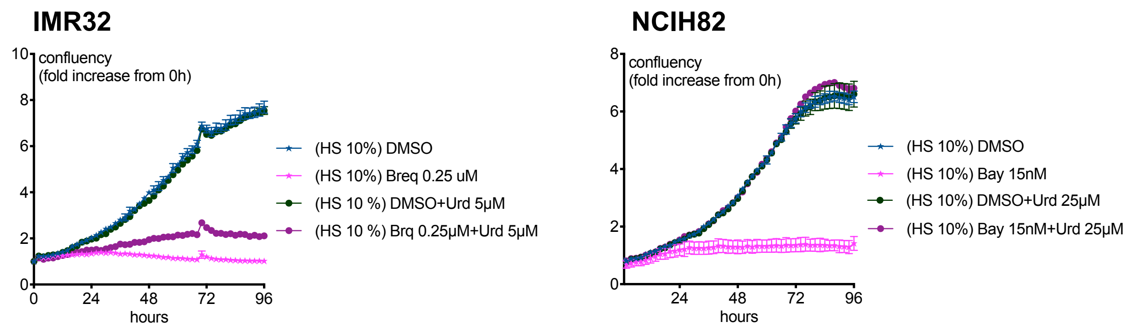

Figure S10. DHODHi are very toxic to cancer cells grown in human serum but can be rescued by adding uridine. Cell confluency was measured with the IncuCyte system.

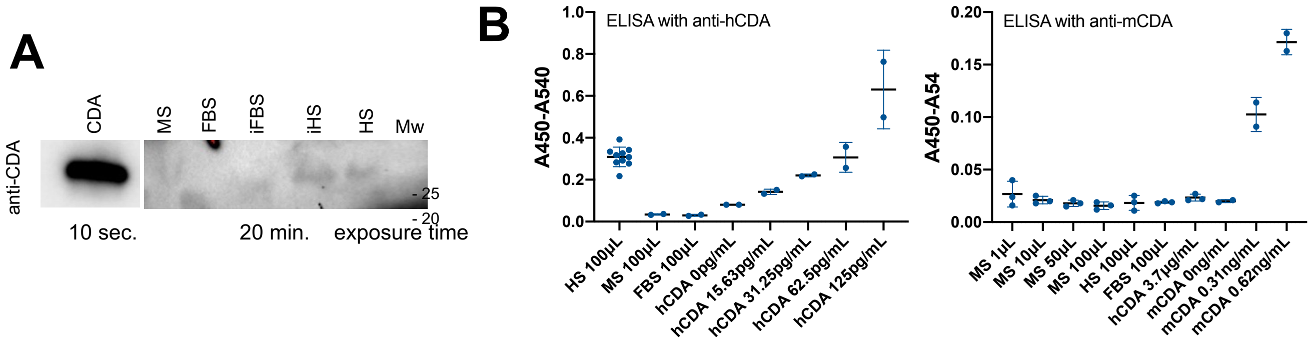

Figure S11. CDA levels in equal volumes of sera measured by western blotting and by ELISA. CDA was not detectable in mouse serum (MS) or fetal bovine serum (FBS) by any method. hCDA and mCDA, recombinant human and mouse CDA, respectively. At present, it is not possible to exclude that the antibodies used against human CDA only recognize human CDA, although the sequence identity between human and mouse CDA is 81.5% and the sequence identity between human and bovine CDA is 80.8%. Note also that the ELISA assay with anti-mouse CDA is less sensitive than with anti-human CDA.

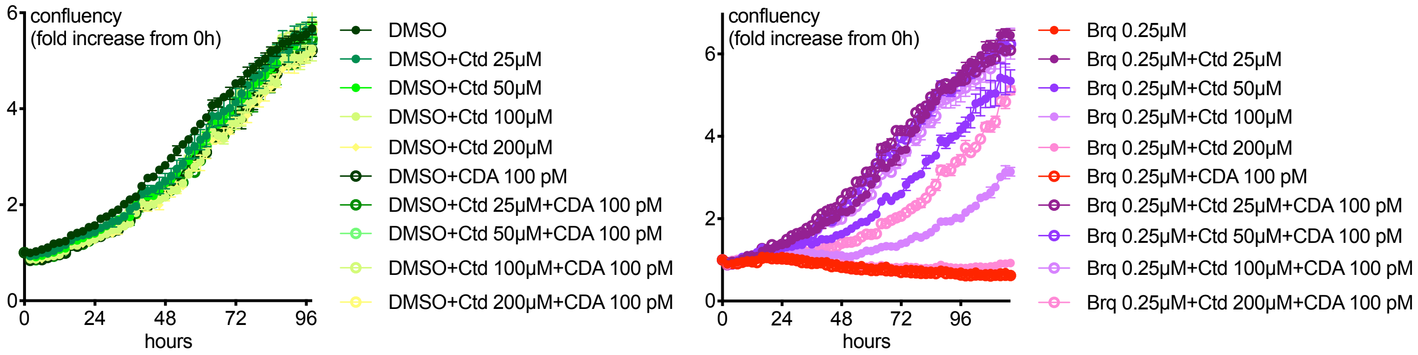

Figure S12. Effect of DHODHi + increasing concentrations of cytidine on IMR32 cells grown in the presence of HS. Cell confluency was measured with the IncuCyte system.

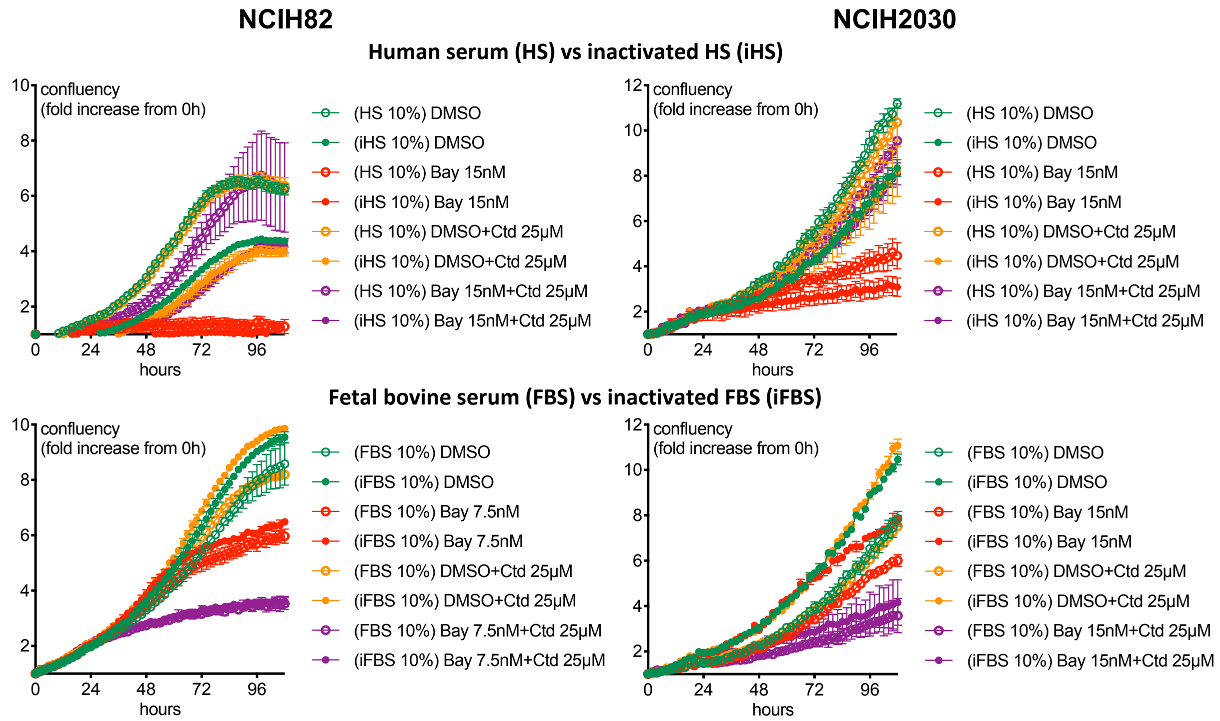

Figure S13. The differences in response to cytidine between HS and FBS are not due to serum inactivation. Cell confluency as well as the proportion of YOYO-3 positive cells were measured with the IncuCyte system.

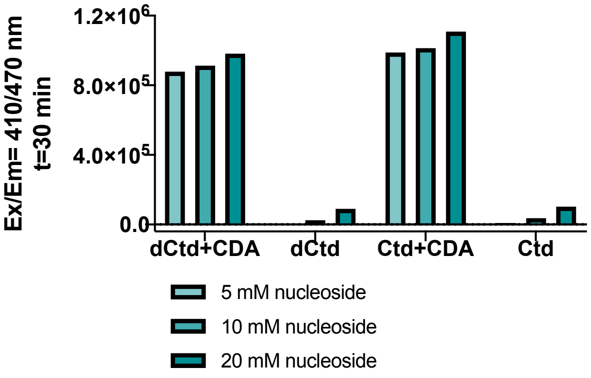

Figure S14. Enzymatic activity assay confirming that like cytidine, deoxycytidine is a substrate for human CDA.

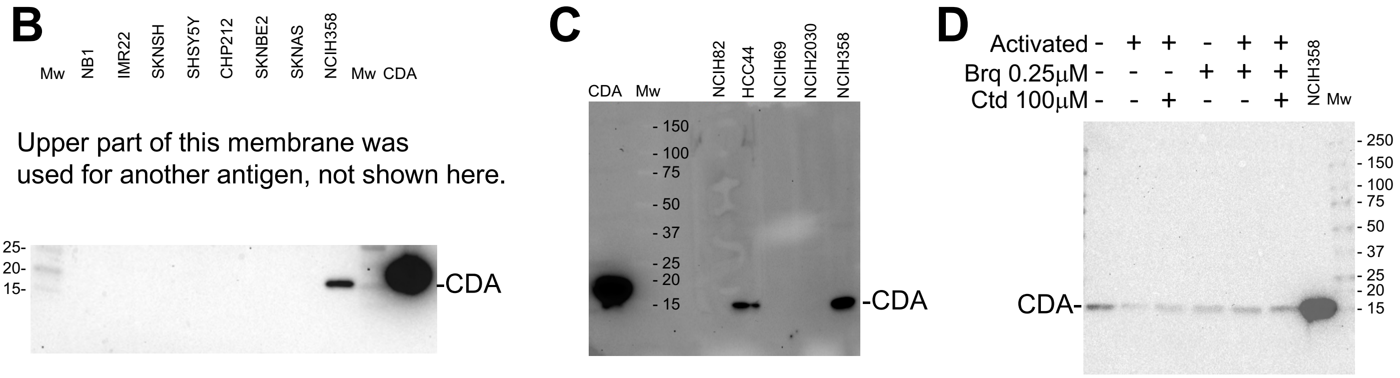

Figure S15. Full size blots in Figure 2.
(B-C) CDA protein expression analyzed by Western blots in neuroblastoma cell lines (B) and lung cancer cell lines (C).
(D) CDA protein levels in non-activated and activated (48h) T cells treated as indicated.

Table S1. CDA gene expression in cell lines from the Cancer Cell Line Encyclopedia 21q4 (CCLE) database available in R2

Malignancy	# cell lines	# cell lines in other malignancies (background)	Mean target (log2[TPM+1])	Mean background (log2[TPM+1])	Welch stat	Welch p-value	FDR
Choriocarcinoma	3	1293	0,141776797	2,126666372	-30,27368461	2,4647E-136	1,4049E-134
Neuroblastoma	28	1268	0,102374631	2,166670709	-29,39061141	7,488E-129	2,1341E-127
Acute Lymphoblastic Leukemia (ALL)	35	1261	0,117760721	2,177702874	-28,98665322	1,9189E-127	3,6459E-126
Hodgkin Lymphoma	8	1288	0,114722383	2,134539728	-29,36688059	6,9959E-102	9,9691E-101
Uveal Melanoma	8	1288	0,041196133	2,134996413	-30,31989579	1,0032E-101	1,1437E-100
Small Cell Lung Carcinoma (SCLC)	51	1245	0,291298258	2,197067259	-18,24006246	1,8742E-38	1,78049E-37
Melanoma	77	1219	0,694164813	2,212267644	-10,92693744	4,47261E-20	3,64198E-19
Non Hodgkin Lymphoma	57	1239	0,3507558	2,20356083	-10,3776714	3,69069E-16	2,62962E-15
Upper Aerodigestive Squamous	55	1241	4,63286652	2,01079556	9,422327137	1,84847E-13	1,1707E-12
Non-Small Cell Lung Carcinoma (NSCLC)	133	1163	3,60058944	1,952989298	7,45184895	5,57398E-12	3,17717E-11
Lymphoma Unspecified	18	1278	0,287065016	2,147916885	-11,5227121	2,58162E-11	1,33775E-10
Rhabdomyosarcoma	17	1279	0,449242086	2,144306359	-10,58906434	2,63727E-10	1,2527E-09
Ewing Sarcoma	16	1280	0,482638613	2,142564634	-9,948017994	2,28571E-09	1,0022E-08
Exocrine	50	1246	4,067326654	2,044011731	5,962623987	2,10499E-07	8,57031E-07
Medulloblastoma	8	1288	0,436057642	2,132543857	-10,96168878	5,34563E-07	2,03134E-06
Chronic Myelogenous Leukemia (CML)	17	1279	0,451808191	2,144272252	-7,273810387	7,07013E-07	2,51873E-06
Glioma	68	1228	1,3289178	2,165992295	-4,293136993	4,60072E-05	0,000154259
Colorectal Adenocarcinoma	68	1228	3,081768967	2,068928876	4,131338022	8,98792E-05	0,000284617
Cholangiocarcinoma	35	1261	3,875180666	2,073412867	4,37228175	0,00010152	0,000304559
Multiple Myeloma	29	1267	1,066996265	2,14622104	-3,675476846	0,000892064	0,002542383
Merkel Cell Carcinoma	3	1293	0,42504686	2,126009133	-10,66765815	0,00216692	0,00588164
Malignant Rhabdoid Tumor	15	1281	0,883872604	2,136570539	-3,516135436	0,00312417	0,008094441
Fibroblast Skin	7	1289	2,977864764	2,117424279	3,503392952	0,010109302	0,024213976
Bladder Carcinoma	36	1260	3,159038791	2,092444089	2,707699013	0,010195358	0,024213976
Osteosarcoma	14	1282	1,254698984	2,131543809	-2,854630711	0,012553848	0,028622773
Esophagus Squamous	25	1271	3,114740821	2,102546364	2,640211391	0,013956775	0,030597545
Unspecified Leukemia	3	1293	0,424512458	2,126010373	-6,378889241	0,017415832	0,036766756
Cervical Adenocarcinoma	3	1293	5,375600437	2,114522929	6,521215614	0,02086433	0,042473816
Chronic Lymphoblastic Leukemia (CLL)	4	1292	0,671783793	2,126561775	-3,24071247	0,045064034	0,0871917
Thyroid Carcinoma	14	1282	3,681829132	2,105038488	2,203406513	0,045890368	0,0871917
Ovary Adenocarcinoma	57	1239	2,692881359	2,095811712	1,968554757	0,05350169	0,098374076
Hepatocellular Carcinoma	22	1274	3,122979179	2,104787604	1,943122464	0,065111576	0,115979995
Endometrial Adenocarcinoma	27	1269	1,432018428	2,136753705	-1,886577906	0,069754015	0,120447118
Renal Cell Carcinoma	31	1265	1,459381062	2,13831157	-1,861691942	0,071845649	0,120447118
Gastric Adenocarcinoma	36	1260	2,850948838	2,101246659	1,787360505	0,082128621	0,133752326
Skin Squamous	4	1292	4,620013694	2,114338153	2,445372108	0,091383388	0,144690364
Cervical Carcinoma	7	1289	4,673424121	2,108216432	1,960509831	0,097394549	0,150040252
Gallbladder Adenocarcinoma	6	1290	4,092558785	2,112906664	1,891321562	0,116721545	0,175082317
Synovial Sarcoma	6	1290	0,867522939	2,127906831	-1,527012508	0,186585102	0,270792367
Breast Ductal Carcinoma	34	1262	1,623588419	2,135501539	-1,336431182	0,190029731	0,270792367
Fibroblast Breast	6	1290	3,251495706	2,116818585	1,374611032	0,226986594	0,315566729
Prostate Adenocarcinoma	9	1287	3,348291111	2,113496759	1,236910659	0,250914692	0,340527081
Fibroblast Lung	6	1290	2,845974617	2,11870473	1,193552174	0,285058763	0,377868593
Cervical Squamous	6	1290	2,714270326	2,119317308	0,874217239	0,42131402	0,545793162
Mesothelioma	20	1276	1,730654885	2,12820678	-0,770364223	0,450262519	0,570332524
Chondrosarcoma	3	1293	1,234075112	2,124132037	-0,841500843	0,488078613	0,604793064
Fibroblast Soft Tissue	3	1293	1,585057869	2,123317692	-0,711925292	0,549417408	0,65154561
Leiomyosarcoma	3	1293	3,05111236	2,119916173	0,711243194	0,550385448	0,65154561
Fibroblast Colorectal	3	1293	2,818478313	2,120455927	0,691743706	0,560100612	0,65154561
Acute Myelogenous Leukemia (AML)	43	1253	1,953690408	2,127850169	-0,547672991	0,586573415	0,668693694
Endometrial Squamous	3	1293	3,241460409	2,11947453	0,561654116	0,630790966	0,705001668
Fibroblast Bone	9	1287	2,016565615	2,122809525	-0,44923973	0,663486101	0,727282841
Esophagus Adenocarcinoma	7	1289	2,535029463	2,119829126	0,397442225	0,704693186	0,754347654
Fibroblast Lymphocyte	3	1293	1,950918152	2,122468828	-0,418221912	0,714645146	0,754347654
Liposarcoma	10	1286	2,309423709	2,120614862	0,24210617	0,814051456	0,843653327
Breast Carcinoma	24	1272	2,024682152	2,123909259	-0,201348457	0,84213545	0,857173583
Thyroid Squamous	3	1293	2,025756783	2,122295188	-0,080638233	0,943034824	0,943034824

Table S2. Copy number (absolute) for relevant 1p genes according to Depmap.

			IMR32	SKNSH	SHSY5Y	CHP212	NB1	SKNBE2	SKNAS
Gene	Chromosome position		Loss 1p32.3-pter	No 1p loss	No 1p loss	Loss 1p13.2-pter	Loss 1p32.2-pter	cnLOH 1p21.3-pter	Loss 1p36.22-36.32
DPYD	1p21.3		3	2	2	1	3	1	2
AK5	1p31.1		3	2	2	1	3	1	2
AK4	1p31.3		3	2	2	1	2	1	2
CMPK1	1p33		1	2	2	1	1	1	2
CTPS1	1p34.2		1	2	2	1	1	1	2
NT5C1A	1p34.2		1	2	2	1	1	1	2
AK2	1p35.1		1	2	2	1	1	1	2
CDA	1p36.12		1	2	2	1	1	1	2
CASP9	1p36.21		1	2	2	1	1	1	2
MTOR	1p36.22		1	2	2	1	1	1	2
CASZ1	1p36.22		1	2	2	1	1	1	1
MIR34A	1p36.22		1	2	2	1	1	1	1
TP73	1p36.32		1	2	2	1	1	1	1
KIF1B	1p36.22		1	2	2	1	1	1	1
CAMTA1	1p36.31		1	2	2	1	1	1	1
CHD5	1p36.31		1	2	2	1	1	1	1

Table S3. Copy number (absolute) for relevant 1p genes according to Depmap.

cell line	1p status	log2(TPM+1)
chp-126	WT	0,111
chp-134	1p_del	0,000
chp-212	1p_del	0,084
gi-me-n	1p_del	0,176
imr-32	1p_del	0,111
kelly	1p_del	0,000
kp-n-rt-bm-1	1p_del	0,098
kp-n-si9s	WT	0,251
kp-n-yn	1p_del	0,000
lan-2	1p_del	0,043
ls	WT	0,043
mhh-nb-11	1p_del	0,163
nb-1	1p_del	0,000
nb1643	1p_del	0,000
ngp	1p_del	0,070
nh-12	1p_del	0,000
nh-6	1p_del	0,124
sh-sy5y	WT	0,000
sima	1p_del	0,098
sk-n-as	1p_del	0,202
sk-n-be(2)	1p_del	0,299
sk-n-dz	WT	0,176
sk-n-fi	WT	0,124
sk-n-sh	WT	0,651
tgw	WT	0,000

Table S4. Cell line information according to Depmap and Cancer Cell Line Encyclopedia (CCLE)

Cell line	Disease	KRAS status	KRAS copy no.	TP53 status	TP53 copy no.	MYC RNAseq Log2(TPM+1)	CDA status	CDA copy no.	CDA proteomics	CDA RNAseq Log2(TPM+1)	CDA Affy (CCLE)	CDADC1 copy no.	CDADC1 RNAseq Log2(TPM+1)
NCIH 358	NSCLC	G12C heteroz.	6	null and wt	2	4.33	wt	4	1,98	6,90	10,98	3	3,07
HCC44		G12C homoz.	7	p.S94*+ p.R175L	7	7.76	wt	7	3,88	5,35	9,38	7	2,71
NCIH 2030		G12C homoz.	2	p.G262V	2	5.56	wt	2	-1,38	0,04	4,10	1	2,9
NCIH82	SCLC	wt	3	Splice site mt p.T125T	3	9.61	wt	3	NA	0,54	4,00	4	3,77
SKNSH	Neuroblastoma	wt	2	wt	2	4.80	wt	2	NA	0,65	4,44	2	3,31
SHSY5Y		wt	2	wt	2	5.58	wt	2	NA	0.00	4,22	2	NA
IMR32		wt	3	wt	2	0.41	wt	1	NA	0,11	3,87	2	3,48
SKNBE2		wt	2	C135F	2	0.50	wt	1	NA	0,30	4,45	2	3,63
SKNAS		wt	2	H168R	1	5.36	wt	2	-1,28	0,20	4,28	2	3,58
NB1		wt	2	wt	1	0.28	wt	1	NA	0,00	4,24	3	4,21
CHP212		wt	2	wt	2	0.71	wt	1	NA	0,08	4,36	2	2,91

Table S5. Properties of neuroblastoma cell lines.

Cell Line	chromosome 1	chromosome 3	chromosome 11	chromosome 17	ALK mutation	p53	MYCN status
SKNAS	Loss p36.22-36.32	Loss p14.2-pter	q13.4-qter	Gain q21.31-qter	WT	H168R	Non-amplified
CHP212	Loss p13.2-pter	Gain/AI p26.3	cnLOH 23.3	Gain q12-qter	WT	WT	Amplified
IMR32	Loss p32.3-pter	Loss p12.3	cnLOH q23.1	Gain q21.2-qter	WT	WT	Amplified
NB1	Loss p32.2-pter	Gain p24.1-pter	cnLOH q23.1	Gain q22-qter	WT; amplified	WT	Amplified
SKNBE(2)	cnLOH p21.3-pter	Loss p14.2-pter	Gain/AI q13.1-qter	Gain q12-qter	WT	C135F	Amplified
SKNSH	None	None	None	Gain q21.31-qter	F1174L	WT	Non-amplified
SHSY5Y	None	None	Loss q22.1-q24.2	Gain q21.31-qter	F1174L	WT	Non-amplified
