# Supplementary material for: Proof-of-principle studies on a strategy to enhance nucleotide imbalance specifically in cancer cells
Source: Cell Death Discov. 2022 Nov 24;8:464. doi: 10.1038/s41420-022-01254-4 (PMC9691752; doi:10.1038/s41420-022-01254-4)
Supplement: Supplementary file 2 — Figure S15: original data [file 41420_2022_1254_MOESM2_ESM.pdf]

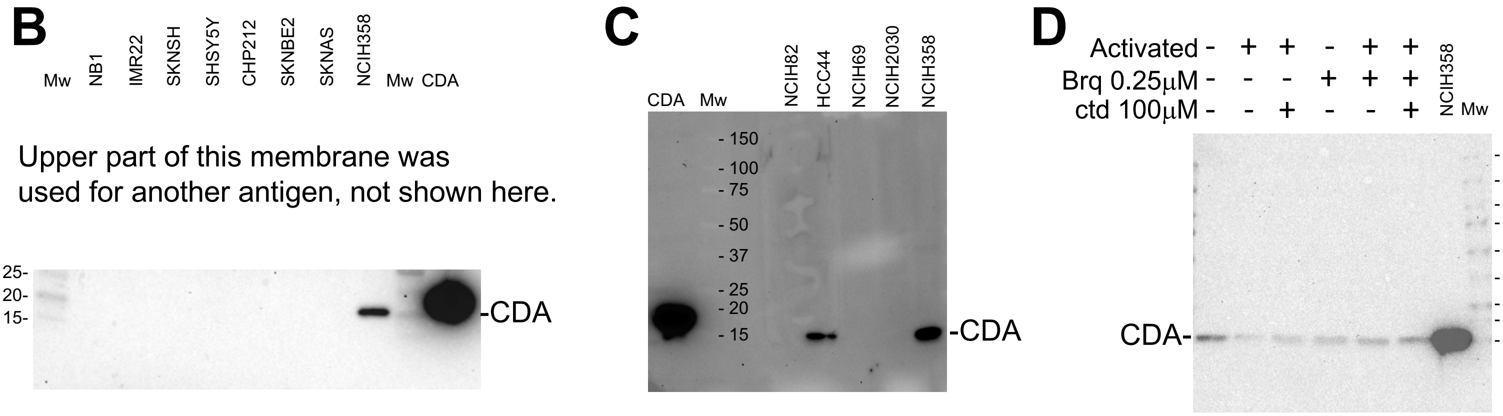

Figure S15. Full size blots in Figure 2.
(B-C) CDA protein expression analyzed by Western blots in neuroblastoma cell lines (B) and lung cancer cell lines (C).
(D) CDA protein levels in non-activated and activated (48h) T cells treated as indicated.
